# The Influence of Aryl Substituents on the Supramolecular Structures and Photoluminescence of Cyclic Trinuclear Pyrazolato Copper(I) Complexes

**DOI:** 10.3390/nano11113101

**Published:** 2021-11-17

**Authors:** Kiyoshi Fujisawa, Mai Saotome, Yoko Ishikawa, David James Young

**Affiliations:** 1 Department of Chemistry, Ibaraki University, Ibaraki 310-8512, Japan; mango4869@gmail.com; 2 Department of Chemistry, Graduate School of Pure and Applied Sciences, University of Tsukuba, Tsukuba 305-8571, Japan; 18s3014l@vc.ibaraki.ac.jp; 3 College of Engineering, IT & Environment, Charles Darwin University, Darwin, NT 0909, Australia; david.young@cdu.edu.au

**Keywords:** polynuclear, copper, crystal structure, pyrazolate ligand, photoluminescence

## Abstract

Cyclic trinuclear complexes with group 11 metal(I) ions are fascinating and important to coordination chemistry. One of the ligands known to form these cyclic trinuclear complexes is pyrazolate, which is a bridging ligand that coordinates many transition metal ions in a Npz–M–Npz linear mode (Npz = pyrazolyl nitrogen atom). In these group 11 metal(I) ions, copper is the most abundant metal. Therefore, polynuclear copper(I) complexes are very important in this field. The cyclic trinuclear copper(I) complex **[Cu(3,5-Ph_2_pz)]_3_** (3,5-Ph_2_pz^–^ = 3,5-diphenyl-1-pyrazolate anion) was reported in 1988 as a landmark complex, but its photoluminescence properties have hitherto not been described. In this study, we report the photoluminescence and two different polymorphs of **[Cu(3,5-Ph_2_pz)]_3_** and its derivative [**Cu(3-Me-5-Phpz)]_3_** (3-Me-5-Phpz^–^ = 3-metyl-5-phenyl-1-pyrazale anion). The substituents in **[Cu(3-Me-5-Phpz)]_3_** cause smaller distortions in the solid-state structure and a red-shift in photoluminescence due to the presence of intermolecular cuprophilic interactions.

## 1. Introduction

Cyclic trinuclear complexes with group 11 metal(I) ions have been of interest to coordination chemists for three decades [1,2,3,4]. One of the ligands known to form these cyclic trinuclear complexes is pyrazolate, which is a bridging ligand that coordinates many transition metal ions in a Npz–M–Npz linear mode (Npz = pyrazolyl nitrogen atom) [5,6,7,8,9,10].

The first structural characterization of the cyclic trinuclear pyrazolato group 11 metal(I) complexes was performed by Fackler and co-workers in 1988 for [Cu(μ-(3,5-Ph_2_pz)]_3_•1/3(*n*-hexane) (3,5-Ph_2_pz^−^ = 3,5-diphenyl-1-pyrazolate anion, denoted here as [Cu(μ-L5pz)]_3_•1/3(*n*-hexane)) [11], quickly followed by a report of the corresponding silver(I) and gold(I) analogues: [Ag(μ-(3,5-Ph_2_pz)]_3_ and [Au(μ-(3,5-Ph_2_pz)]_3_ [12]. Since this pioneering research, many cyclic trinuclear pyrazolato group 11 metal(I) complexes have been prepared [1,2,3,4,5,6,7,8,9,10]. In the present work, we investigated new copper(I) complexes, because of the abundance of this metal [13]. Table 1 lists the known cyclic trinuclear pyrazolato copper(I) complexes bridged by aryl- and alkyl-substituted pyrazololates and the new complex reported herein. Many trinuclear pyrazolato copper(I) complexes have been prepared with varying substituents [11,14,15,16,17,18,19,20,21,22,23,24,25,26]. In this series, the central nine-membered Cu_3_N_6_ rings are almost planar, with N–Cu–N bond angles ranging from 169.2(3) to 179.4(6)°, indicating that each copper(I) ion is two-coordinate in a nearly linear arrangement with the two pyrazolyl nitrogen atoms. The three copper(I) ions form a triangle with bond angles ranging from 57.15 to 63.19(2)°, and intramolecular bond distances from 3.1078(7) to 3.272 Å, which is slightly longer than twice the Cu Bondi’s van der Waals radius (2.80 Å = 1.40 Å × 2) [27], indicating only very weak cuprophilic (Cu⋯Cu) intramolecular interactions. Many cyclic trinuclear pyrazolato copper(I) complexes, with the notable exception of **[Cu(μ-L5pz)]_3__,_** have intermolecular cuprophilic interactions between each trinuclear plane and Cu⋯Cu distances ranging in length from 2.9099(4) to 4.3155 Å [28,29,30]. Similar metallophilic interactions have been observed in other trinuclear complexes of group 11 metal(I) ions [1,2,3,4,5,6,7,8,9].

**Figure 1:**
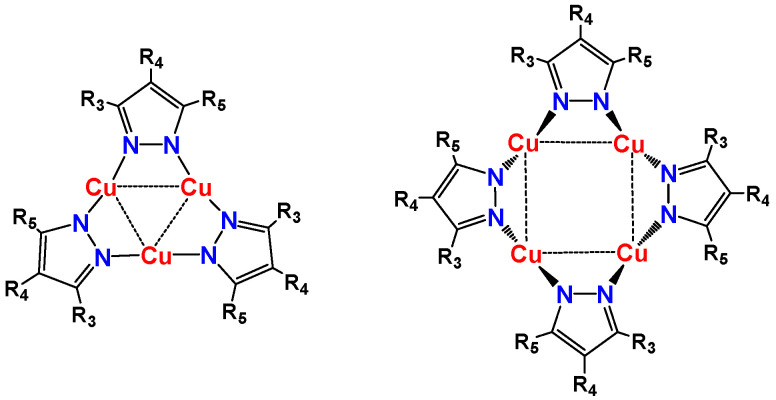
Tri- and tetranuclear pyrazolato copper(I) complexes showing intramolecular Cu···Cu interactions.

We have previously reported pyrazolato copper(I) complexes synthesized from alkyl-substituted pyrazoles to form cyclic trinuclear and tetranuclear structures, depending on the nature of the substituents on the pyrazolate ring (Figure 1) [18,19,30]. Bulky substituents in the 3,5-positions resulted in tetranuclear pyrazolato copper(I) complexes [Cu(μ-3-tBu-5-iPrpz)]_4_ and [Cu(μ-3,5-tBu_2_pz)]_4_ [18,19]. A tetranuclear pyrazolato copper(I) complex [Cu(μ-L5pz)]_4_ was formed from the ligand L5pz-H [31].

**Figure 2:**
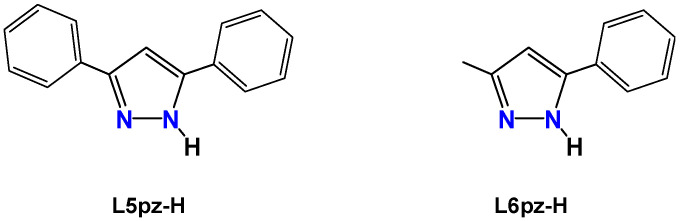
Pyrazoles used in this research.

In the present work, we report the preparation and physicochemical properties of the known **[Cu(μ-L5pz)]_3_** complex (Figure 2) but obtained as different polymorphs from different crystallization solvents. We then investigated the substituent effects in new cyclic trinuclear copper(I) complex of 3-metyl-5-phenyl-1-pyrazale (denoted as L6pz-H) (Figure 2) [32].

## 2. Materials and Methods

### 2.1. Material and Techniques

All complexes were prepared under an argon atmosphere using standard Schlenk tube techniques. Dichloromethane and acetonitrile were dried by distillation from phosphorous pentoxide and calcium hydride, respectively, under an argon atmosphere. Diethyl ether and *n*-heptane were distilled from sodium benzophenone ketyl under argon [33]. Ultra-dry acetone was purchased and deoxygenated with a stream of argon. Deuterochloroform was obtained from Cambridge Isotope Laboratories, Inc (Andover, MA, USA). Other reagents were commercially available and were used without further purification. The 3,5-diphenyl-1-pyrazole (L5pz-H) [34] and 3-methyl-5-phenyl-1-pyrazole (L6pz-H) [32] were prepared by published methods. The complex [Cu(μ-3,5-iPr_2_pz)]_3_ was also prepared by a published method [19,20]. Sodium pyrazolate anions were prepared from the corresponding pyrazoles using one equivalent of sodium hydride in diethyl ether (room temperature, 1–2 h).

### 2.2. Instrumentation

IR spectra (4000–400 cm^–1^) and far-IR spectra (680–150 cm^–1^) were acquired as KBr pellets using a JASCO FT/IR-6300 spectrophotometer and as CsI pellets using a JASCO FT/IR 6700 spectrophotometer (JASCO, Tokyo, Japan), respectively. Raman spectra (4000–200 cm^–1^) were obtained on solid powders employing a JASCO RFT600 spectrophotometer fitted with a YAG laser 600 mW (JASCO, Tokyo, Japan). ^1^H-NMR (500 MHz) spectra were obtained on a Bruker AVANCE III-500 NMR spectrometer at room temperature (298 K) in CDCl_3_ as solvent (Bruker Japan, Yokohama, Japan). ^1^H chemical shifts were reported as *δ* values relative to residual chloroform. UV-Vis spectra (solution and solid, 800–200 nm) were recorded on a JASCO V-570 spectrophotometer (JASCO, Tokyo, Japan). The values of *ε* were calculated per metal(I) ion. Mulls for spectroscopy were prepared by finely grinding microcrystalline material into powders with a mortar and pestle and then adding mulling agents (nujol, poly(dimethylsiloxane), viscosity 10,000). Fluorescence spectra were acquired on a JASCO FP-6500 (solid, 700–300 nm) spectrofluorometer (JASCO, Tokyo, Japan). Absorption and luminescence spectra were recorded using solid samples cooled with a liquid nitrogen cryostat (CoolSpeK USP-203, Unisoku Scientific Instruments, Osaka, Japan). Elemental analyses were performed at the Department of Chemistry, University of Tsukuba.

### 2.3. Preparation of Complexes

#### 2.3.1. [Cu(μ-L5pz)]_3_

NaL5pz was synthesized using NaH (38.1 mg, 1.588 mmol) and L5pz-H (383.3 mg, 1.74 mmol). This solution of NaL5pz in ether (40 cm^3^) was added to a solution of CuCl (173.4 mg, 1.75 mmol) in acetone (30 cm^3^). The mixture was stirred overnight, and the solvent was evaporated under vacuum. The resulting solid was extracted with dichloromethane (30 cm^3^) to remove NaCl. Recrystallization of the resulting colorless solid from dichloromethane/heptane at −30 °C provided colorless crystals (67.6 mg, 0.08 mmol, 15%) that were filtered and dried under vacuum. The colorless crystals were obtained by recrystallization from dichloromethane for **[Cu(μ-L5)]_3_**•**2(CH_2_Cl_2_)**. Colorless crystals of **[Cu(μ-L5)]_3_** were obtained by recrystallization from dichloromethane/acetonitrile.

Calcd. for C_45_H_33_Cu_3_N_6_·2/3(CH_2_Cl_2_): C, 60.60; H, 3.82; N, 9.29. Found: C, 60.25; H, 3.73; N, 9.36. 

IR (KBr, cm^−1^): 3061 m (C–H), 3033 m (C–H), 1942 w, 1869 w, 1800 w, 1604 m, 1538 w, 1513w, 1472 s (C=N), 1447 w, 1429 w, 1406 w, 1338 m, 1154 w, 1115 w, 1071 w, 1006 m, 910 m, 751 s, 714 m, 693 s, 556 m. 

Far-IR (CsI, cm^−1^): 66 7s, 618 w, 556 s, 479 m, 471 m, 441 w, 403 w, 373 w, 313 w, 275 w, 228 m, 207 m. 

Raman (solid, cm^−1^): 3130 w, 3057 m, 1605 s, 1537 m, 1515 m, 1473 w, 1428 m, 1406 m, 1341 w, 1323 w, 1295 w, 1225 w, 1180 w, 1156 w, 1118 w, 1030 w, 1002 s, 967 m, 836 w, 765 w, 717 w, 694 w, 669 w, 619 w, 543 w, 528 w, 451 w, 404 w, 360 w, 275 w, 248 w. 

^1^H-NMR (CDCl_3_, 500 MHz): *δ*/ppm (assignments): 6.75 (s, 3H, pz 4-H), 7.05 (t, 12H, 7 Hz, pz*Ph m*), 7.18 (t, 6H, 7 Hz, pz*Ph p*), 7.68 (d, 12H, 7 Hz, pz*Ph o*). 

UV-Vis (solution, dichloromethane, λ_max_/nm(ε/cm^−1^ mol^−1^ dm^3^)) 251 (106,000).

UV-Vis (solid, nujol, nm): 248, 280 (sh).

Emission at 280 nm excitation wavelength (solid, λ_max_/nm): 298 K, 312, 355, 432, 456, 618, 696; 173 K, 312, 347, 430, 472, 509, 616, 681; 83 K, 313, 343, 431, 458, 470, 490, 510, 536, 665.

#### 2.3.2. **[Cu(μ-L6pz)]_3_**

NaL6pz was synthesized using NaH (21.0 mg, 0.875 mmol) and L6pz-H (152.6 mg, 0.96 mmol). This solution of NaL6pz in ether (15 cm^3^) was added to a solution of CuCl (94.8 mg, 0.96 mmol) in acetone (25 cm^3^). The mixture was stirred for 3 days, and the solvent was evaporated under vacuum. The resulting solid was extracted with dichloromethane (30 cm^3^) to remove NaCl. Recrystallization of the resulting colorless from dichloromethane/heptane at −30 ˚C provided colorless crystals (81.5 mg, 0.12 mmol, 41%) that were filtered and dried under vacuum. Colorless crystals suitable for XRD were obtained by recrystallization from dichloromethane/heptane. 

Calcd. for C_30_H_27_Cu_3_N_6_·1/6(CH_2_Cl_2_): C, 53.57; H, 4.07; N, 12.43. Found: C, 53.81; H, 4.05; N, 12.57.

IR (KBr, cm^−1^): 3063 m (C–H), 2979 m (C–H), 2917 m (C–H), 1941 w, 1873 w, 1799 w, 1605 m, 1533 s, 1501 s (C=N), 1477 s, 1448 s, 1411 s, 1342 s, 1263 m, 1138 m, 1075 m, 1031 w, 984 w, 903 w, 755 s, 735 s, 693 s, 536 w, 437 w. 

Far-IR (CsI, cm^−1^): 660 m, 649 m, 618 w, 578 w, 540 s, 487 s, 439 s, 372 w, 281 w, 223 s. 

Raman (solid, cm^−1^): 3121 w (C–H), 3061 m (C–H), 2923 w (C–H), 1604 s, 1532 s (C=N), 1430 s, 1343 w, 1204 w, 1157 w, 1077 w, 1102 m, 986 m, 733 w, 651 w, 620 w, 406 w, 370 w. 

^1^H-NMR (CDCl_3_, 500 MHz): *δ*/ppm (assignments): 2.31 (s, 9H, pz*Me*), 6.33 (s, 3H, pz 4-H), 7.29 (s, br, 9H, pz*Ph m* and *p*), 7.79 (s, br, 6H, pz*Ph o*). 

UV-Vis (solution, dichloromethane, λ_max_/nm(ε/cm^−1^ mol^−1^ dm^3^)): 255 (55,500). UV-Vis (solid, nujol, nm): 211, 298. 

Emission at 280 nm excitation wavelength (solid, λ_max_/nm): 298 K, 344, 434, 463, 485, 582, 606, 652; 173 K, 338, 386sh, 433, 462, 481, 518, 578, 650; 83 K, 333, 388, 433, 445, 475, 569.

### 2.4. X-ray Crystal Structures

Crystal data and corresponding refinement parameters for **[Cu(μ-L5)]_3_**, **[Cu(μ-L5)]_3_•2(CH_2_Cl_2_)**, and **[Cu(μ-L6)]_3_•0.5(CH_2_Cl_2_)** are given in Table 2 (CCDC numbers: 25117510 for **[Cu(μ-L5)]_3_**, 25117511 for **[Cu(μ-L5)]_3_•2(CH_2_Cl_2_)**, and 25117512 for **[Cu(μ-L6)]_3_•0.5(CH_2_Cl_2_)**).

The diffraction data for **[Cu(μ-L5)]_3_•2(CH_2_Cl_2_)** were measured on a Rigaku/MSC Mercury CCD system (Rigaku, Tokyo, Japan) with graphite monochromated Mo Kα (λ = 0.71070 Å) radiation at −70 °C. A suitable crystal was coated with Paratone-N oil and mounted on a glass fiber. The unit cell for each crystal was determined using CrystalClear [35]. The diffraction data of **[Cu(μ-L5)]_3_** and **[Cu(μ-L6)]_3_•0.5(CH_2_Cl_2_)** were measured on a Rigaku XtaLAB P200 diffractometer using monochromated Mo Kα (λ = 0.71075 Å) radiation at −95 °C (Rigaku, Tokyo, Japan). A suitable crystal was coated with Paratone-N oil and mounted on a Dual-Thickness MicroLoop LD (200 μM). The highly redundant data sets were reduced using CrysAlisPro [36]. Structures were solved by direct methods (SIR2008 [37] and SIR2004 [38]). Refinement was carried out by a full matrix least-squares method on *F*^2^ using the CrystalStructure [39] crystallographic software package, except for refinement, which was performed using SHELXL 2013 [40]. Hydrogen atoms were placed in calculated positions (Table 2).

## 3. Results and Discussion

### 3.1. Synthesis

**Figure 3:**
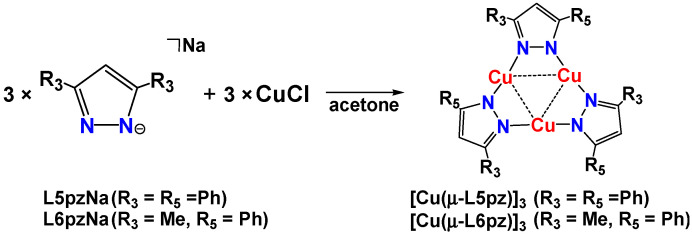
Synthesis of cyclic trinuclear pyrazolato copper(I) complexes.

Cyclic trinuclear pyrazolato copper(I) complexes have previously been prepared using copper metal [21], copper(I) oxide [20,25,26], copper(I) salts [11,15,18,19,22,23,24], or from copper(II) salts [14,16,17]. We employed CuCl as the copper source (Figure 3) and obtained complexes that were unstable even in the solid-state. The color of the powders or crystals rapidly changed from colorless to brown when exposed to air at room temperature. 

### 3.2. Structure

**Figure 4:**
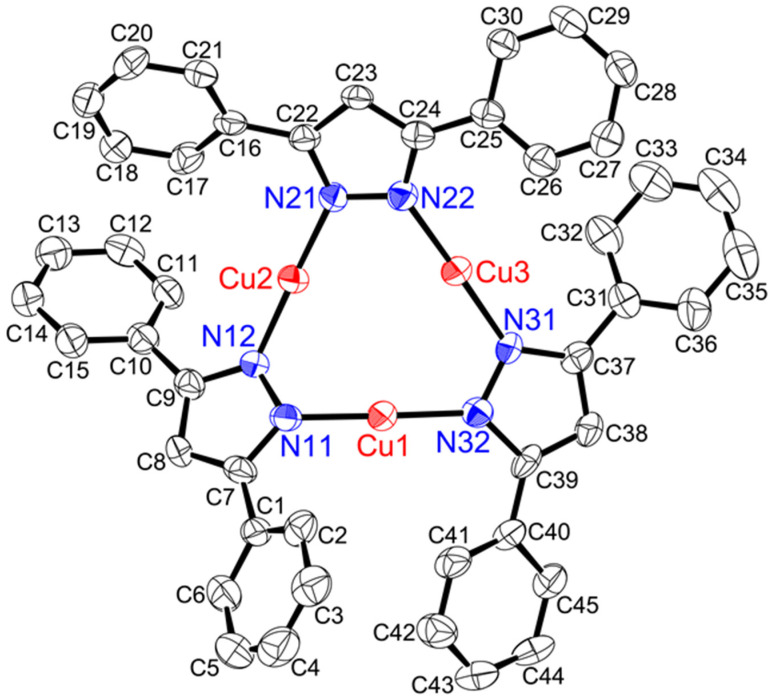
Crystal structure of **[Cu(μ-L5pz)]_3_** showing 50% displacement ellipsoids and the atom labeling scheme. Hydrogen atoms are omitted for clarity. Relevant bond lengths (Å) and angles (°): Cu1–N11, 1.864(5); Cu1–N32, 1.852(5); Cu2–N12, 1.857(6); Cu2–N21, 1.855(5); Cu3–N22, 1.867(5); Cu3–N31, 1.864(5); N11–Cu1–N32, 177.3(3); N12–Cu2–N21, 175.2(3); N22–Cu3–N31, 177.6(2); Cu1···Cu2, 3.1567(11), Cu2···Cu3, 3.1727(12), Cu3···Cu1, 3.1563(12); Cu1···Cu2···Cu3, 59.82(3); Cu2···Cu3···Cu1, 59.84(3); Cu3···Cu1···Cu2, 60.34(3).

**Figure 5:**
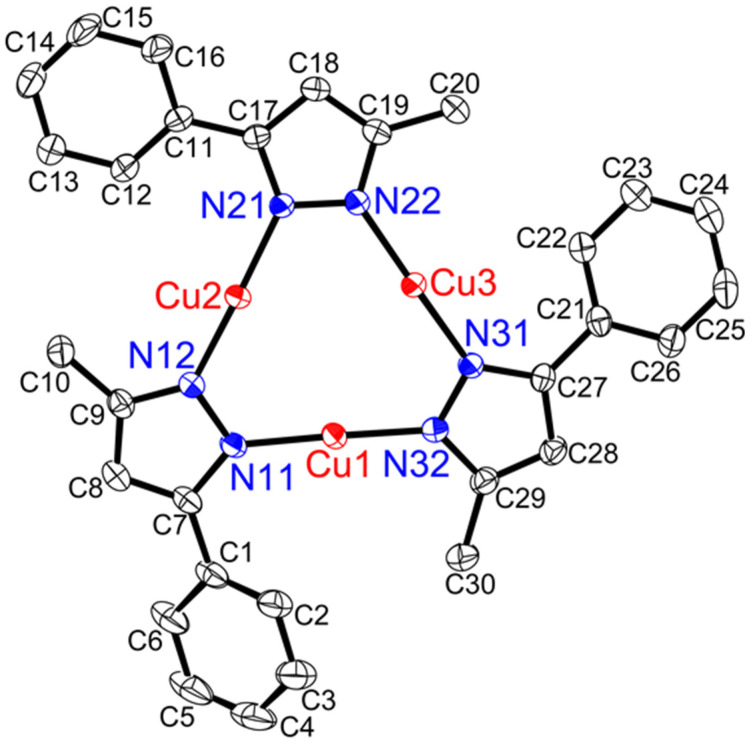
Crystal structure of **[Cu(μ-L6pz)]_3_** showing 50% displacement ellipsoids and the atom labeling scheme. Two crystallographically independent molecules are present, whose structural features are essentially identical. Molecule 1 is presented here. Hydrogen atoms are omitted for clarity. Relevant bond lengths (Å) and angles (°): Cu1–N11, 1.8629(18); Cu1–N32, 1.8574(17); Cu2–N12, 1.866(2); Cu2–N21, 1.869(2); Cu3–N22, 1.865(2); Cu3–N31, 1.864(2); N11–Cu1–N32, 177.14(11); N12–Cu2–N21, 173.46(8); N22–Cu3–N31, 175.35(8); Cu1···Cu2, 3.1867(5), Cu2···Cu3, 3.2452(4), Cu3···Cu1, 3.1848(5); Cu1···Cu2···Cu3, 59.352(10); Cu2···Cu3···Cu1, 59.410(10); Cu3···Cu1···Cu2, 61.238(10).

Single-crystal X-ray structural analysis was performed on [**Cu(μ-L5pz)]_3_**, **[Cu(μ-L5pz)]_3_•2(CH_2_Cl_2_)**, and two polymorphs of **[Cu(μ-L6)pz]_3_•0.5(CH_2_Cl_2_)** (Figure 4, Figure 5, Figures S1 and S2), respectively. The relevant bond lengths (Å) and angles (°) are noted in their captions. **[Cu(μ-L6)pz]_3_•0.5(CH_2_Cl_2_)** was obtained as two crystallographically independent molecules, whose structural features were essentially identical. Their packing diagrams are given in Figure S3 for [**Cu(μ-L5pz)]_3_**, Figure S4 for **[Cu(μ-L5pz)]_3_•2(CH_2_Cl_2_)**, Figure S5 for **[Cu(μ-L6)pz]_3_•0.5(CH_2_Cl_2_)**, and Figure S6 for [Cu(μ-L5pz)]_3_•1/3(*n*-hexane) [11].

[**Cu(μ-L5pz)]_3_** (Figure 4) and **[Cu(μ-L5pz)]_3_•2(CH_2_Cl_2_)** (Figure S1) existed as trinuclear structures without any intermolecular cuprophilic interaction and with three intramolecular cuprophilic distances, which were slightly longer than twice the Bondi’s van der Waals radius (2.80 Å = 1.40 Å × 2) [27]. Both structures were very close to the structure of the original complex [Cu(μ-L5pz)]_3_•1/3(*n*-hexane) [11]. By comparison, **[Cu(μ-L6)pz]_3_•0.5(CH_2_Cl_2_)** (Figure 5 and Figure S2) existed as a trinuclear structure with three intramolecular cuprophilic interactions and two weak intermolecular cuprophilic interactions between each cyclic trinuclear plane, which were 2.9099(4) Å (molecule 1, symmetry operators: −X + 1, −Y + 2, −Z + 1) and 2.9622(4) Å (molecule 2, symmetry operators: −X, −Y + 1, −Z), forming the hexanuclear structure {**[Cu(μ-L6)pz]_3_**}_2_ (Figure S5). Complexes [**Cu(μ-L5pz)]_3_**, **[Cu(μ-L5pz)]_3_•2(CH_2_Cl_2_)**, and [Cu(μ-L5pz)]_3_•1/3(*n*-hexane) did not have any intermolecular interactions between each trinuclear structure [11] (Table 1, and Figures S3–S6). This is unusual for cyclic trinuclear complexes [1,3,5]. Generally, such solid-state structures have one or two intermolecular cuprophilic interactions to form a hexanuclear structure (Table 1) [5]. This difference between **[Cu(μ-L5pz)]_3_** and other complexes may be due to the planarity of the nine-membered Cu_3_N_6_ plane. The torsion angles (Cu–Npz–Npz–Cu) of **[Cu(μ-L5)]_3_** were 49.2(5), 11.0(6), 12.7(6)°, and those of **[Cu(μ-L5pz)]_3_•2(CH_2_Cl_2_)** and [Cu(μ-L5pz)]_3_•1/3(*n*−hexane) were 11.8(3), 7.0(4), 40.9(3)° and 5.6, 39.6(6), 7.0(7)°, respectively (Table 1). Therefore, one of the torsion angles of the **[Cu(μ-L5pz)]_3_** complex is approximately 40°, which is unusual for cyclic trinuclear copper(I) structures. Most torsion angles in such complexes are less than 15°. This distortion also resulted in contact distances for Cu1⋯N12 of 2.719(5) Å in [**Cu(μ-L5pz)]_3_** and Cu1⋯N12 of 2.747(3) Å in **[Cu(μ-L5pz)]_3_•2(CH_2_Cl_2_)**. These short distances are also unprecedented. The same large torsion angles in **[Cu(μ-L6)pz]_3_•0.5(CH_2_Cl_2_)** were 10.8(2) and 6.2(2)° in [Cu(μ-3,4-Ph_2_pz)]_3_ 18.8(8) and 12.8(7)° [14], in [Cu(μ-3-Phpz)]_3_ 10.1(8)° [15], in [Cu(μ-3,5-iPr_2_pz)]_3_ 13.0(3)° [18,19] or 13.9(1)° [20] in [Cu(4-Ph-μ-3,5-Me_2_pz)]_3_•1/2(CHCl_3_) 10.3 and 10.6° [22], and in [Cu(μ-3-CF_3_-5-Phpz)]_3_ 24.9(2)° [20]. The larger substituents such as phenyl and isopropyl groups influence these torsion angles.

This distortion in [**Cu(μ-L5pz)]_3_** also affected its crystal packing (Figures S3, S4 and S6). [**Cu(μ-L5pz)]_3_** exhibited irregular arrangements in its packing diagram with some larger spaces without any pseudo dimeric structures, as previously observed for other trinuclear copper(I) complexes bearing intermolecular cuprophilic interactions (Table 1). These voids in [**Cu(μ-L5pz)]_3_** contained solvent molecules that varied with crystallization conditions and solvents. By contrast, **[Cu(μ-L6)pz]_3_•0.5(CH_2_Cl_2_)** had two intermolecular cuprophilic interactions, 2.9099(4) Å (molecular 1) and 2.9622(4) Å (molecule 2), to form a hexanuclear structure (Figure S5).

### 3.3. Solution-State Properties

The ^1^H-NMR spectra of the obtained white powders **[Cu(μ-L5pz)]_3_** and **[Cu(μ-L6pz)]_3_** were measured in CDCl_3_ (Figures S7 and S8, respectively) and chemical shifts and assignments are listed in the Material and Methods section. Intermolecular interactions in their solid-state are not present upon dissolution, converting to the known cyclic trinuclear copper(I) complexes with chemical shifts’ distinct from those of free ligand. This observation is also supported by solution-state UV-Vis spectra (see Section 2.3). The absorption band at 251 nm for **[Cu(μ-L5pz)]_3_** and 255 nm for **[Cu(μ-L6pz)]_3_** were clearly shifted from the absorption bands of the corresponding pyrazole ligand (data not shown). The molar absorbance of **[Cu(μ-L5pz)]_3_** at 251 nm was almost twice that of **[Cu(μ-L6pz)]_3_** at 255 nm, due to the presence of twice as many phenyl rings (Figure S9).

### 3.4. Solid-State Properties

IR and Raman spectra of **[Cu(μ-L5pz)]_3_** and **[Cu(μ-L6pz)]_3_** are reproduced in Figures S10–S15. The C=N stretching vibration appeared at 1472 cm^−1^ (IR) in **[Cu(μ-L5pz)]_3_** and 1501 cm^−1^ (IR) in **[Cu(μ-L6pz)]_3_**. This assignment was confirmed by comparison of the same C=N stretching vibrations at 1523 cm^−1^ (IR) for [Cu(3,5-iPr_2_pz}_3_] [19]. The Cu–N stretching vibration could be assigned to 556 cm^−1^ (IR) and was observed at 542 cm^−1^ and 528 cm^−1^ (Raman) in **[Cu(μ-L5pz)]_3_**, which compares well with the values of 539 cm^−1^ (IR) and 562 cm^−1^ and 527 cm^−1^ (Raman) in **[Cu(μ-L6pz)]_3_**. This assignment was also confirmed by comparison with the Cu–N stretching vibration energy at 501 cm^−1^ (IR) and 482 cm^−1^ (Raman) in [Cu(3,5-iPr_2_pz}_3_] [19]. The distortion in **[Cu(μ-L5pz)]_3_** resulted in C=N, and Cu–N stretching energies there were weaker than those in **[Cu(μ-L6pz)]_3_**.

The solid-state UV-Vis absorption spectra of **[Cu(μ-L5pz)]_3_** and **[Cu(μ-L6pz)]_3_** acquired as a Nujol suspension are shown in Figure S16. The characteristic absorption bands at 225 nm and 280 (sh) nm in **[Cu(μ-L5pz)]_3_** and 211 nm and 298 nm in **[Cu(μ-L6pz)]_3_** were obviously shifted to the lower energy side from the corresponding solution-state absorption maxima at 251 nm in **[Cu(μ-L5pz)]_3_** and 255 nm in **[Cu(μ-L6pz)]_3_**. This shift in both **[Cu(μ-L5pz)]_3_** and **[Cu(μ-L6pz)]_3_** may have been caused by very weak intermolecular interactions in the solid-state packing (Figures S3–S6). For their detailed assignment, density functional theory calculations are required, which is beyond the scope of this article. Nevertheless, these absorption bands can be assigned to a copper(I) to pyrazolate charge transfer (MLCT) based on the other reported polynuclear copper(I) complexes [1,3,4,19,20].

**Figure 6:**
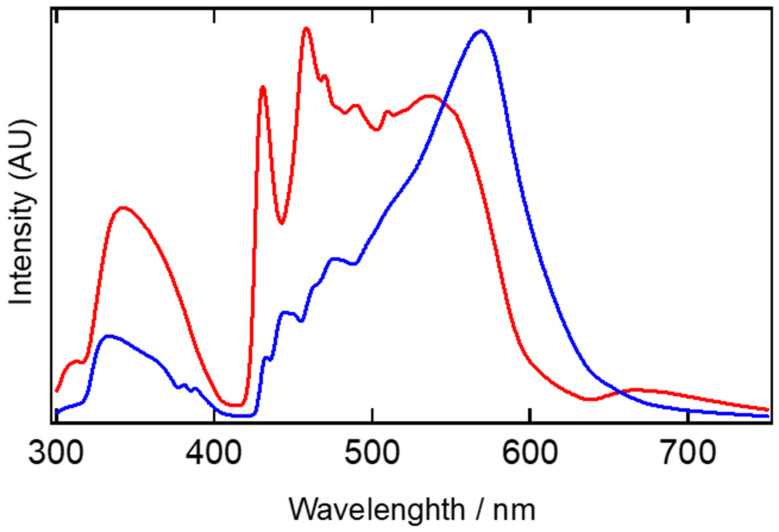
Solid-state photoluminescence spectra of **[Cu(μ-L5)]_3_** (red line) and **[Cu(μ-L6)]_3_** (blue line) at 83 K recorded at 280 nm excitation.

The solid-state photoluminescence spectra of **[Cu(μ-L5)]_3_** and **[Cu(μ-L6)]_3_** at 83 K excited at 280 nm are shown in Figure 6. The temperature-dependent photoluminescent spectra in **[Cu(μ-L5)]_3_** and **[Cu(μ-L6)]_3_** were recorded at 298 K and 173 K. (Figures S17 and S18). The corresponding variable temperature emission spectra for [Cu(μ-3,5-iPr_2_pz)]_3_ are also shown in Figure S19 [18,19].

In the 83 K experiments, **[Cu(μ-L6)]_3_** had a strong emission band at 569 nm and a weak emission band at 333 nm. In addition, some vibrational structures derived from the phenyl ring were observed around 450 nm (445, 475, and 475 nm). In **[Cu(μ-L5)]_3_**, however, the emission band at 536 nm was very weak, and some vibrational structures derived from the phenyl ring at around 460 nm (431, 458, 470, and 490 nm) were stronger. Of course, there are twice the number of phenyl rings in **[Cu(μ-L5)]_3_**; however, the intensity of the emission band at 536 nm was very weak compared to that at 313 nm. This may be due to the absence of intermolecular cuprophilic interactions, which weakened the emission band at 536 nm.

To confirm this observation, we examined the emission of [Cu(μ-3,5-iPr_2_pz)]_3_ (Figure S17). Since there are no phenyl rings, no vibrational structures were observed, and this molecule had two intermolecular cuprophilic interactions (3.0250(7) Å). The emission band at 551 nm was very strong at low temperatures, suggesting that the emission band at 500–600 nm was due to intermolecular cuprophilic interactions. These lower energy emissions are attributed to metal-based phosphorescence arising from closed shell d^10^−d^10^ intermolecular cuprophilic interactions [1,3,4,19,20]. We are now in the process of probing the origin of this photoluminescent behavior by theoretical and more detailed physicochemical measurements.

## 4. Conclusions

Cyclic trinuclear copper(I) complexes of **[Cu(μ-L5pz)]_3_** and **[Cu(μ-L6)pz]_3_** were synthesized and their structure and physicochemical properties examined. The introduction of a phenyl group in the pyrazole ring distorted the solid-state trinuclear structure with torsion angles (Cu–Npz–Npz–Cu) in **[Cu(μ-L5pz)]_3_** complexes around 40°. By comparison, torsion angles in the reported cyclic trinuclear copper(I) structures were similar to those in **[Cu(μ-L6)pz]_3_•0.5(CH_2_Cl_2_)** and less than 15°. This distortion in [**Cu(μ-L5pz)]_3_** also resulted in irregular arrangements in the packing diagram with some larger spaces and without any pseudo dimeric structures from intermolecular cuprophilic interactions. These voids were easily occupied by solvent molecules giving different crystal structures from different crystallization conditions and solvents.

The ^1^H-NMR spectra of the obtained white powder **[Cu(μ-L5pz)]_3_** and **[Cu(μ-L6pz)]_3_** in CDCl_3_ and UV-Vis spectra indicated that intermolecular interactions in their solid-state were not stable upon dissolution, converting to known cyclic trinuclear copper(I) complexes. Solid-state IR (mid and far region) and FT-Raman spectroscopy revealed that both the C=N and Cu–N stretching energies of **[Cu(μ-L5pz)]_3_** were weaker than those in **[Cu(μ-L6pz)]_3_** due to this distorted structure and absence of cuprophilic interactions.

The solid-state UV-Vis absorption spectra of **[Cu(μ-L5pz)]_3_** and **[Cu(μ-L6pz)]_3_** exhibited copper(I) to pyrazolate charge transfer (MLCT) around 250 nm. Excitation in the solid-state at 280 nm generated a lower energy photoluminescence band at 536 nm in **[Cu(μ-L5pz)]_3_** and at 569 nm in **[Cu(μ-L6pz)]_3_**. We assigned these bands to metal-based phosphorescence arising from closed shell d^10^–d^10^ intermolecular cuprophilic interactions based on the precedent in the literature. Further efforts to probe how the structure and photoluminescence of cyclic trinuclear copper(I) complexes are affected by the coordination environment are in progress.

## Figures and Tables

**Table 1:** Structural parameters of selected cyclic trinuclear pyrazolato copper(I) complexes.

Complex	*d* Intra (Cu⋯Cu) /Å	∠ Cu⋯Cu⋯Cu /Deg	*d* Inter (Cu⋯Cu) /Å ^a^	∠ Npz−Cu−Npz ^b^ /Deg	Torsion Angle ^b^ ∠ Cu–Npz–Npz–Cu/Deg	Ref.
**[Cu(μ-L5)]_3_**	3.1567(11), 3.1727(12), 3.1563(12)	59.82(3), 59.84(3), 60.34(3)	n.a.	177.3(3), 175.2(3), 177.6(2)	−49.2(5), 11.0(6), 12.7(6)	this work
**[Cu(μ-L5)]_3_** **•2(CH_2_Cl_2_)**	3.1078(7), 3.1247(6), 3.2651(8)	63.19(2), 58.16(2), 58.66(2)	n.a.	173.5(2), 176.9(2), 176.4(2)	11.8(3), 7.0(4), −40.9(3)	this work
**[Cu(μ-L6)]_3_** **•0.5(CH_2_Cl_2_)**	3.1867(5), 3.2452(4), 3.1848(5), 3.2063(5), 3.2344(4), 3.1946(4)	59.35(1), 59.41(1), 61.24(1), 59.47(1), 59.83(1), 60.70(1)	2.9099(4), 2.9622(4)	177.1(1), 173.5(1), 175.4(1), 177.8(1), 174.6(1), 171.9(1)	0.8(2), 4.4(2), 0.5(2), 10.8(2), −6.2(2), −3.3(2)	this work
[Cu(μ-L5)]_3_ •1/3(C_6_H_14_)	3.280(1), 3.406(1), 3.332(1)	62.02, 59.74, 58.24	n.a.	178.6(3), 169.6(3), 169.2(3)	5.6, −39.6(6), 7.0(7)	[11]
[Cu(μ-3,4-Ph_2_pz)]_3_	3.147, 3.259, 3.213, 3.325, 3.138, 3.235	60.18, 58.19, 61.63, 59.99, 62.86, 57.15	3.483(2), 3.483(2), 2.921(1), 2.921(1)	177.9(3), 177.6(3), 174.0(3), 171.6(3), 174.0(3), 175.4(3)	0.0(8), −4.9(8), −7.3(7), 18.8(8), 12.8(7), 0.6(8)	[14]
[Cu(μ-3-Phpz)]_3_	3.2076, 3.2083, 3.2402	59.66, 60.67, 59.68	3.099(2), 3.559(2)	177.8(3), 178.5(3), 173.6(3)	0.7(8),−10.1(8), −0.7(8)	[15]
[Cu(μ-3,5-Me_2_pz)]_3_	3.207, 3.197, 3.257, 3.195, 3.204, 3.258	61.15, 59.57, 59.28, 61.22, 59.25, 59.53	2.944(2), 2.947(2)	173.4(2), 175.3(2) 173.5(2), 174.7(2) 175.2(2), 173.6(2)	−0.7(6), 0.8(6), 0.4(6), −1.0(5), 0.2(6), −0.3(6)	[16]
[Cu(μ-3,5-Me_2_pz)]_3_	3.1950, 3.2582, 3.2061,	59.57, 59.23, 61.20	2.9534(6), 2.9534(6)	175.4(1), 173.9(1), 173.8(1)	0.9(3), 0.3(3), 0.5(3)	[17]
[Cu(μ-3,5-iPr_2_pz)]_3_	3.1907(6), 3.1997(7), 3.2370(6)	59.43(1), 59.70(1), 60.87(1)	3.0250(7), 3.0250(7)	169.6(1), 171.4(1), 176.9(1)	−4.9(3), 13.0(3), 1.7(3)	[18,19]
[Cu(μ-3,5-iPr_2_pz)]_3_	3.195, 3.211, 3.235	59,42, 59.92, 60.66	2.989, 2.989	169.84, 171.24, 176.88	1.4(1), 13.9(1), −6.1(1)	[20]
[Cu(μ-3,4,5-Me_3_pz)]_3_	3.155, 3.272, 3.207	59.84, 58.27, 61.89	3.069(1), 3.069(1)	174.6(2), 173.9(2), 174.7(2)	3.2(5), 3.0(6), 5.4(5)	[21]
[Cu(4-Ph-μ-3,5-Me_2_pz)]_3_ •1/2(CHCl_3_)	3.284, 3.214, 3.322	61.49, 60.29, 58.23	3.671(1), 3.494(1)	178.7(2), 176.4(2), 176.3(2)	−10.3(4), 6.1(4),−10.6(4)	[22]
[Cu(4-I-μ-3,5-Me_2_pz)]_3_	3.214, 3.180, 3.202	60.10, 60.47, 59.42	3.897(4), 3.631(4)	179.4(6), 177.9(5), 175.5(5)	−9(1), 2(1), 5(1)	[23]
[Cu(4-NO_2_-μ-3,5-Me_2_pz)]_3_	3.185, 3.255, 3.185	59.2, 59.2, 61.7	3.329(7), 3.329(7)	177.6, 177.4(7), 177.4(7)	8, 8(2), 8	[24]
[Cu(μ-3-CF_3_pz)]_3_	3.216, 3.246, 3.231, 3.214, 3.248, 3.264	59.99, 59.54, 60.46, 60.68, 59.14, 60.18	3.100(2), 3.345(1)	177.9(3), 174.5(3), 176.8(3), 178.2(3), 177.9(3), 178.1(3)	5.9(9), −7.4(9), 2.1(8), −0.6(8), −0.6(8), −7.2(8)	[20]
[Cu(μ-3-CF_3_-5-Mepz)]_3_	3.2052, 3.2009, 3.2451	60.87, 59.63, 59.50	3.7040(5), 3.9150(6)	178.7(1), 178.1(1), 179.0(1)	1.9(3), 1.4(3), −3.8(3)	[20]
[Cu(μ-3-CF_3_-5-Phpz)]_3_	3.2197, 3.1473, 3.2580	61.54, 60.32, 58.14	3.8482	175.8(1), 175.3(1), 174.2(1)	−8.2(2), 6.5(2), 24.9(2)	[20]
[Cu(μ-3,5-(CF_3_)_2_pz)]_3_	3.232, 3.242, 3.221	59.67, 60.01, 60.33	3.879, 3.893	179.2(2), 179.0(2), 178.7(2)	2.6(6), 2.8(6), −4.7(6)	[25]
[Cu(μ-3,5-(CF_3_)_2_pz)]_3_	3.2309, 3.2184, 3.2474	60.47, 59.96, 59.57	3.813(1), 3.987(1)	178.4(1), 178.6(1), 178.4(1)	−7.0(3), 4.2(3), 6.3(3)	[20,26]

^a^ n.a. means not available. ^b^ Npz means pyrazolyl nitrogen.

**Table 2:** Crystal data and structure refinement of **[Cu(μ-L5pz)]_3_**, **[Cu(μ-L5pz)]_3_•2(CH_2_Cl_2_)**, and **[Cu(μ-L6)pz]_3_•0.5(CH_2_Cl_2_)**.

Complex	[Cu(μ-L5pz)]_3_	[Cu(μ-L5pz)]_3_ •2(CH_2_Cl_2_)	[Cu(μ-L6pz)]_3_ •0.5(CH_2_Cl_2_)
CCDC number	2,117,510	2,117,511	2,117,512
Empirical formula	C_45_H_33_Cu_3_N_6_	C_47_H_37_Cl_4_Cu_3_N_6_	C_30.5_H_28_Cl_1_Cu_3_N_6_
Formula weight	848.43	1018.30	704.69
Crystal system	Monoclinic	Monoclinic	Triclinic
Space group	*P*2_1_/*c* (#14)	*P*2_1_/*n* (#14)	$P\bar{1}$ (#2)
*a*/Å	12.7491(8)	13.5299(4)	14.65231(15)
*b*/Å	15.9322(11)	14.4842(4)	15.23581(19)
*c*/Å	18.4769(13)	22.9784(7)	15.50482(13)
*α*/°	90	90	117.8200(10)
*β*/°	100.173(6)	105.293(3)	103.3700(8)
*γ*/°	90	90	98.7900(10)
*V*/Å^3^	3694.0(4)	4343.6(2)	2838.24(7)
*Z*	4	4	4
*D*_calc_/g cm^−3^	1.525	1.557	1.649
*μ*(MoKα)/cm^−1^	17.539	17.433	23.542
Temperature/°C	−95.0	−70.0	−95.0
2*θ* range/°	6–55	6–55	6–55
Reflections collected	56,293	34,206	92,558
Unique reflections	8478	9949	13,014
*R*_int_	0.1526	0.0428	0.0284
Number of variables	487	541	730
Refls./Para ratio	17.41	18.39	17.83
Residuals: *R*1 (*I* > 2 σ (*I*))	0.0747	0.0588	0.301
Residuals: *R* (All refl.)	0.1763	0.0805	0.0322
Residuals: *wR2* (All refl.)	0.1568	0.1789	0.858
Goodness of fit ind.	1.035	1.026	1.039
Max/min peak,/e Å^−3^	0.62/−0.54	2.08/−1.67	2.32/−1.08

*R*1 = Σ||*Fo*| − |*Fc*||/] Σ|*Fo*|, *wR2 =* [Σ (*w* (*Fo*^2^ − *Fc*^2^)^2^)/Σ*w*(*Fo*^2^)^2^]^1/2^.

## Data Availability

Not applicable.

## Supplementary Materials

The following are available online at https://www.mdpi.com/article/10.3390/nano11113101/s1, CIFs and checkCIF reports. Figure S1: Structure of **[Cu(μ-L5pz)]_3_•2(CH_2_Cl_2_)**, Figure S2: Structure of **[Cu(μ-L6pz)]_3_** (molecule 2), Figure S3: Packing diagram of crystal structure of **[Cu(μ-L5pz)]_3_**, Figure S4: Packing diagram of **[Cu(μ-L5pz)]_3_•2(CH_2_Cl_2_)**, Figure S5: Packing diagram of **[Cu(μ-L6pz)]_3_•0.5(CH_2_Cl_2_)**, Figure S6: Packing diagram of [Cu(μ-L5pz)]_3_•1/3(*n*-hexane), Figure S7: ^1^H-NMR spectrum of **[Cu(μ-L5pz)]_3_**, Figure S8: ^1^H-NMR spectrum of **[Cu(μ-L6pz)]_3_**, Figure S9: UV-Vis spectra of **[Cu(μ-L5pz)]_3_** and **[Cu(μ-L6pz)]_3_** in dichloromethane, Figure S10: IR spectra of **[Cu(μ-L5pz)]_3_** in KBr disk, Figure S11: IR spectra of **[Cu(μ-L6pz)]_3_** in KBr disk, Figure S12: FT-Raman spectrum of **[Cu(μ-L5pz)]_3_**, Figure S13: FT-Raman spectrum of **[Cu(μ-L6pz)]_3_**, Figure S14: FT-Raman and far-IR spectra of **[Cu(μ-L5pz)]_3_**, Figure S15: FT-Raman and far-IR spectra of **[Cu(μ-L6pz)]_3_**, Figure S16: UV-Vis spectra of **[Cu(μ-L5pz)]_3_** and **[Cu(μ-L6pz)]_3_** in solid mull, Figure S17: Photoluminescence spectra of **[Cu(μ-L5pz)]_3_** solid, Figure S18: Photoluminescence spectra of **[Cu(μ-L6pz)]_3_** solid, Figure S19: Photoluminescence spectra of [Cu(μ-3,5-iPr_2_pz)]_3_ solid.
