# Supplementary material for: The Influence of Aryl Substituents on the Supramolecular Structures and Photoluminescence of Cyclic Trinuclear Pyrazolato Copper(I) Complexes
Source: Nanomaterials (Basel). 2021 Nov 17;11(11):3101. doi: 10.3390/nano11113101 (PMC8624218; doi:10.3390/nano11113101)
Supplement: Supplementary file 1 [file nanomaterials-11-03101-s001.zip › nanomaterials-1460519-supplementary.pdf]

Supplementary Material

The Influence of Aryl Substituents on the Supramolecular Structures and Photoluminescence of Cyclic Trinuclear Pyrazolato Copper(I) Complexes

Kiyoshi Fujisawa [1,2,*], Mai Saotome [1], Yoko Ishikawa [2] and David James Young [3]

[1] Department of Chemistry, Ibaraki University, Ibaraki 310-8512, Japan; mango4869@gmail.com
[2] Department of Chemistry, Graduate School of Pure and Applied Sciences, University of Tsukuba, Tsukuba 305-8571, Japan; 18s3014l@vc.ibaraki.ac.jp
[3] College of Engineering, IT & Environment, Charles Darwin University, Darwin, NT 0909, Australia; david.young@cdu.edu.au
* Correspondence: kiyoshi.fujisawa.sci@vc.ibaraki.ac.jp; Tel.: +81-29-853-8373

Contents

**Figure S1**	Crystal structure of **[Cu(μ-L5pz)]$_3$•2(CH$_2$Cl$_2$)**.	S-1
**Figure S2**	Crystal structure of **[Cu(μ-L6pz)]$_3$** (molecule 2).	S-2
**Figure S3**	Packing diagram of **[Cu(μ-L5pz)]$_3$**.	S-3
**Figure S4**	Packing diagram of **[Cu(μ-L5pz)]$_3$•2(CH$_2$Cl$_2$)**.	S-4
**Figure S5**	Packing diagram of **[Cu(μ-L6pz)]$_3$•0.5(CH$_2$Cl$_2$)**.	S-5
**Figure S6**	Packing diagram of [Cu(μ-L5pz)]$_3$•1/3(*n*-hexane).	S-6
**Figure S7**	^{1}H-NMR spectrum of **[Cu(μ-L5pz)]$_3$** in CDCl$_3$ at room temperature.	S-7
**Figure S8**	^{1}H-NMR spectrum of **[Cu(μ-L6pz)]$_3$** in CDCl$_3$ at room temperature.	S-7
**Figure S9**	UV-Vis spectra of **[Cu(μ-L5pz)]$_3$** and **[Cu(μ-L6pz)]$_3$** in dichloromethane.	S-8
**Figure S10**	IR spectra of **[Cu(μ-L5pz)]$_3$** in KBr disk.	S-9
**Figure S11**	IR spectra of **[Cu(μ-L6pz)]$_3$** in KBr disk.	S-9
**Figure S12**	FT-Raman spectrum of **[Cu(μ-L5pz)]$_3$**.	S-10
**Figure S13**	FT-Raman spectrum of **[Cu(μ-L6pz)]$_3$**.	S-10
**Figure S14**	FT-Raman and far-IR spectra of **[Cu(μ-L5pz)]$_3$**.	S-11
**Figure S15**	FT-Raman and far-IR spectra of **[Cu(μ-L6pz)]$_3$**.	S-11
**Figure S16**	UV-Vis spectra of **[Cu(μ-L5pz)]$_3$** and **[Cu(μ-L6pz)]$_3$** in solid mull.	S-12
**Figure S17**	Photoluminescence spectra of **[Cu(μ-L5pz)]$_3$** solid.	S-13
**Figure S18**	Photoluminescence spectra of **[Cu(μ-L6pz)]$_3$** solid.	S-14
**Figure S19**	Photoluminescence spectra of [Cu(μ-3,5-iPr$_2$pz)]$_3$ solid.	S-15

References S-16

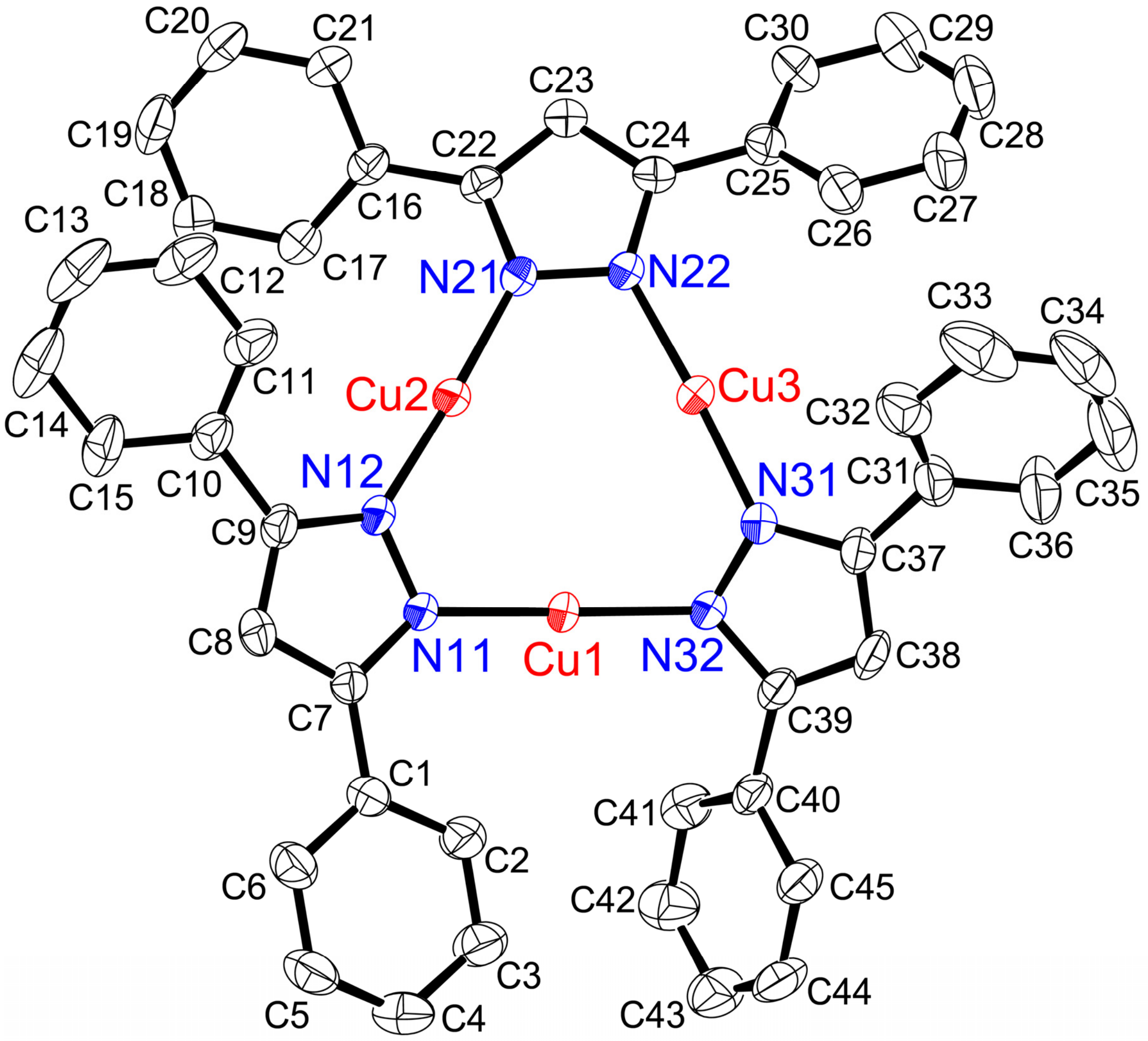

Figure S1. Crystal structure of **[Cu(μ-L5pz)]$_3$•2(CH_2Cl_2)** showing 50% displacement ellipsoids and the atom labeling scheme. Hydrogen atoms and solvent molecules are omitted for clarity. Relevant bond lengths (Å) and angles (°): Cu1–N11, 1.867(3); Cu1–N32, 1.867(4); Cu2–N12, 1.854(4); Cu2–N21, 1.850(4); Cu3–N22, 1.867(4); Cu3–N31, 1.862(4); N11–Cu1–N32, 173.45(14); N12–Cu2–N21, 176.86(15); N22–Cu3–N31, 176.43(16); Cu1···Cu2, 3.1078(7), Cu2···Cu3, 3.1247(6), Cu3···Cu1, 3.2651(8); Cu1···Cu2···Cu3, 63.185(16); Cu2···Cu3···Cu1, 58.155(16); Cu3···Cu1···Cu2, 58.660(16).

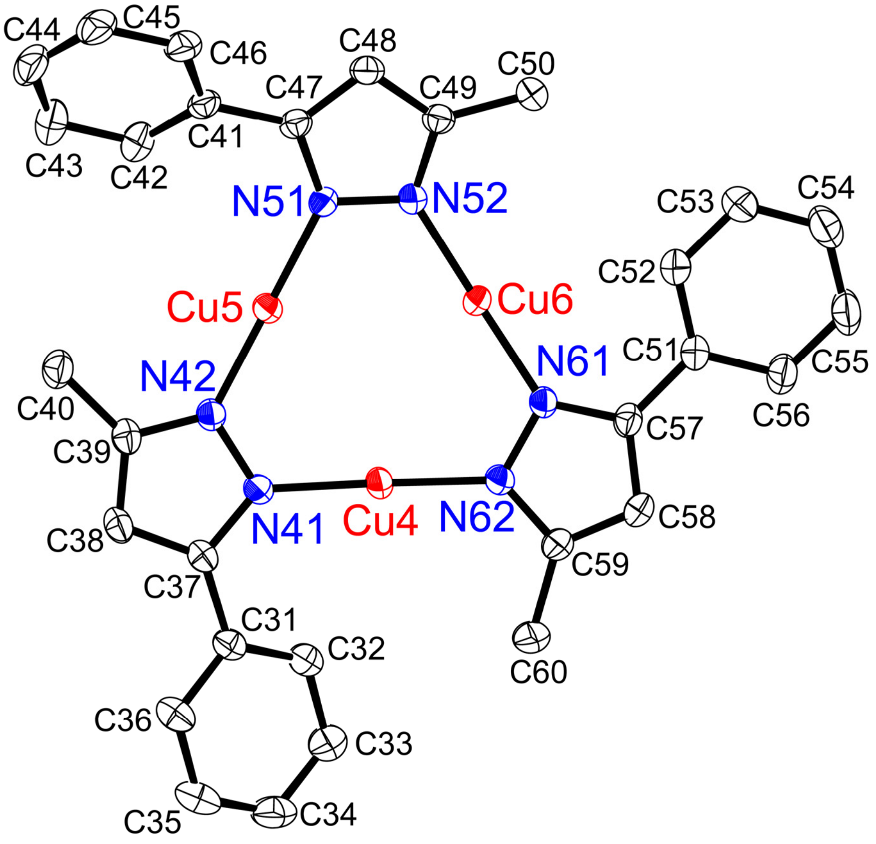

Figure S2. Crystal structure of **[Cu(μ-L6pz)]₃** showing 50% displacement ellipsoids and the atom labeling scheme. Two crystallographically independent molecules are present, whose structural features are essentially identical. Molecule 2 is presented here. Hydrogen atoms are omitted for clarity. Relevant bond lengths (Å) and angles (°): Cu4–N41, 1.8673(18); Cu4–N62, 1.8623(19); Cu5–N42, 1.862(2); Cu5–N51, 1.868(2); Cu6–N52, 1.870(2); Cu6–N61, 1.874(2); N41–Cu1–N62, 177.78(8); N42–Cu2–N51, 174.58(8); N52–Cu6–N61, 171.87(9); Cu4···Cu5, 3.2063(5), Cu5···Cu6, 3.2344(4), Cu6···Cu4, 3.1946(4); Cu4···Cu5···Cu6, 59.468(10); Cu5···Cu6···Cu4, 59.828(10); Cu6···Cu4···Cu5, 60.703(10).

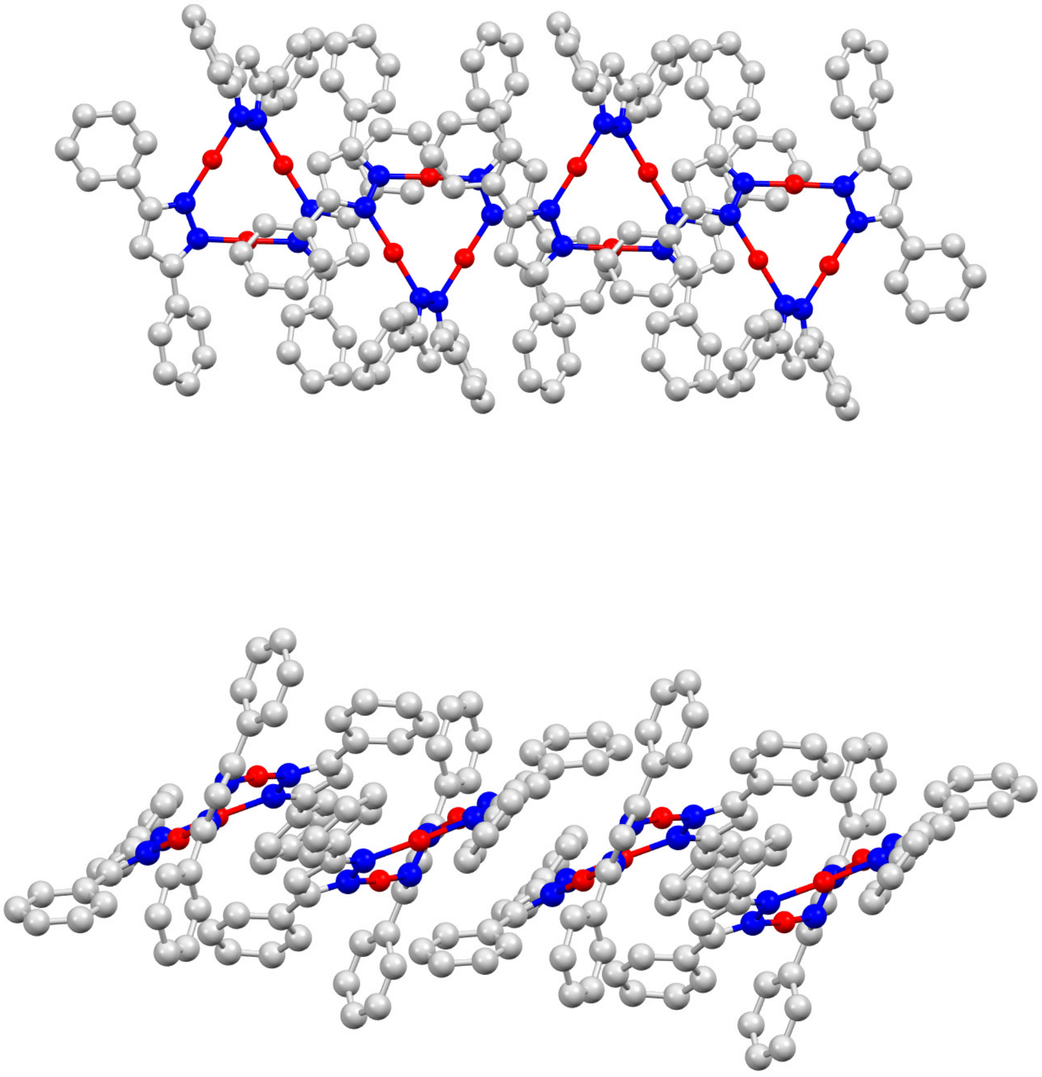

Figure S3. Packing diagram of **[Cu(μ-L5pz)]$_3$** (upper) top view and (bottom) side view. Hydrogen atoms are omitted for clarity. Color: red, copper; blue, nitrogen; grey, carbon. Intermolecular Cu···Cu distances: 5.295, 5.295, 5.475 Å

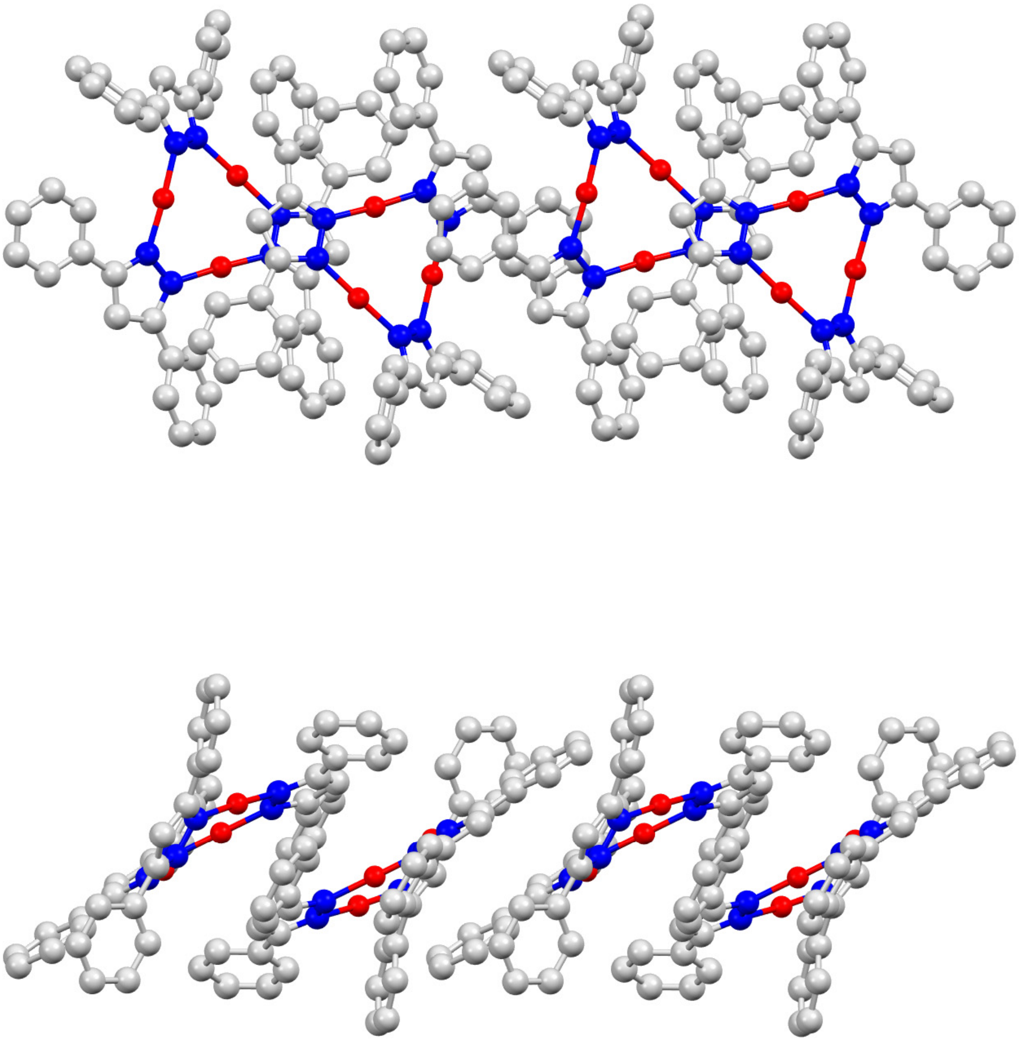

Figure S4. Packing diagram of **[Cu(μ-L5pz)]$_3$•2(CH$_2$Cl$_2$)** (upper) top view and (bottom) side view. Hydrogen atoms and solvents are omitted for clarity. Color: red, copper; blue, nitrogen; grey, carbon. Intermolecular Cu···Cu distances: 5.078, 5.078, 5.691 Å

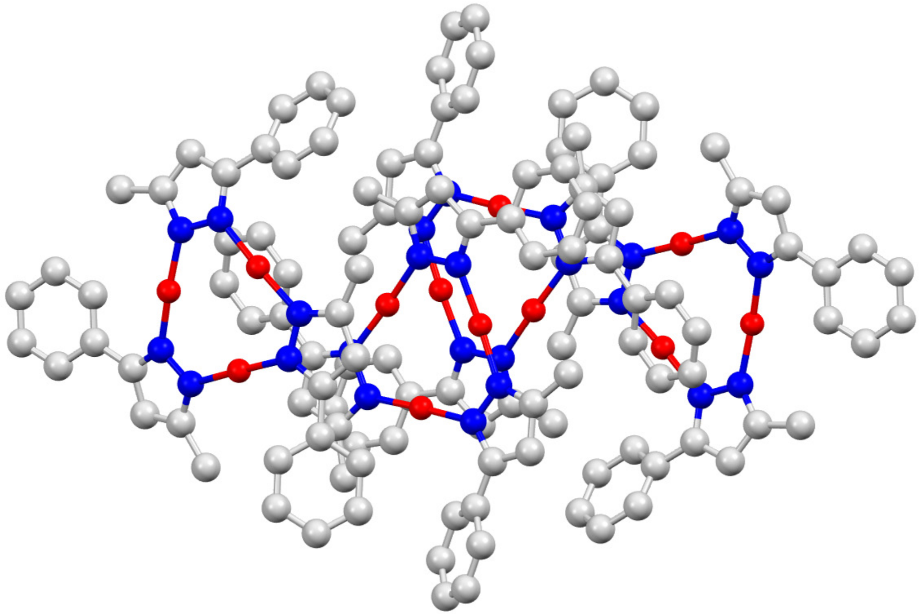

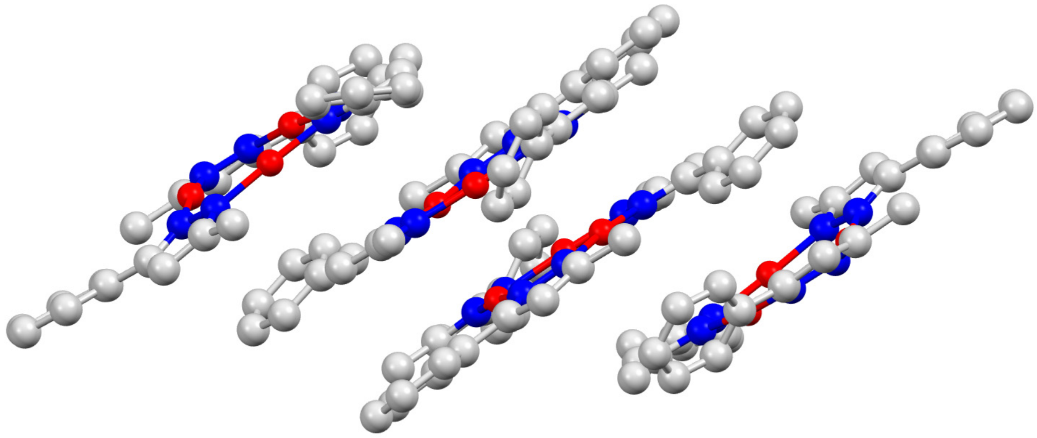

Figure S5. Packing diagram of **[Cu(μ-L6pz)]$_3$•0.5(CH_2Cl_2)** (upper) top view and (bottom) side view. Hydrogen atoms and solvents are omitted for clarity. Color: red, copper; blue, nitrogen; grey, carbon. Intermolecular Cu···Cu distances: 2.9099(4) and 2.9622(4).

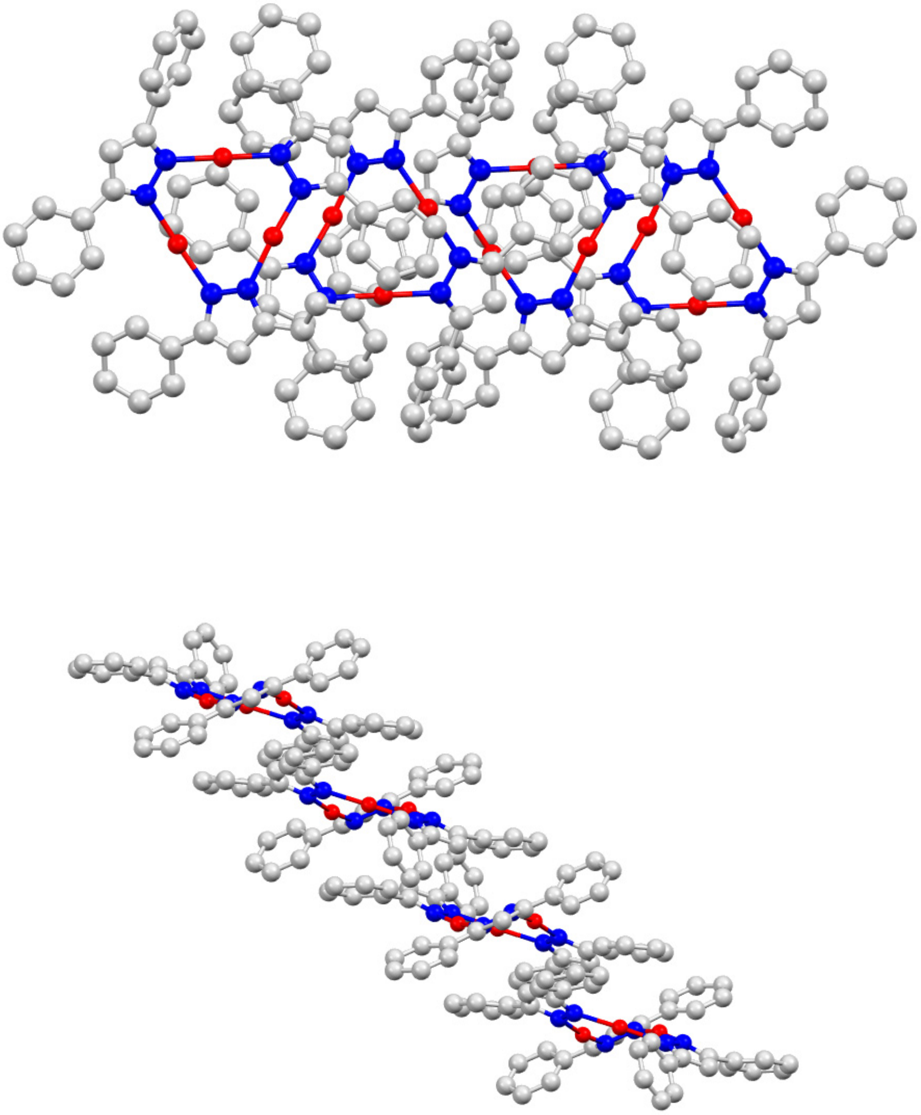

Figure S6. Packing diagram of [Cu(μ-L5pz)]$_3$•1/3(*n*-hexane) (upper) top view and (bottom) side view. Hydrogen atoms and solvents are omitted for clarity. Color: red, copper; blue, nitrogen; grey, carbon. Intermolecular Cu···Cu distances: 5.400, 6.577, 6.577 Å [1].

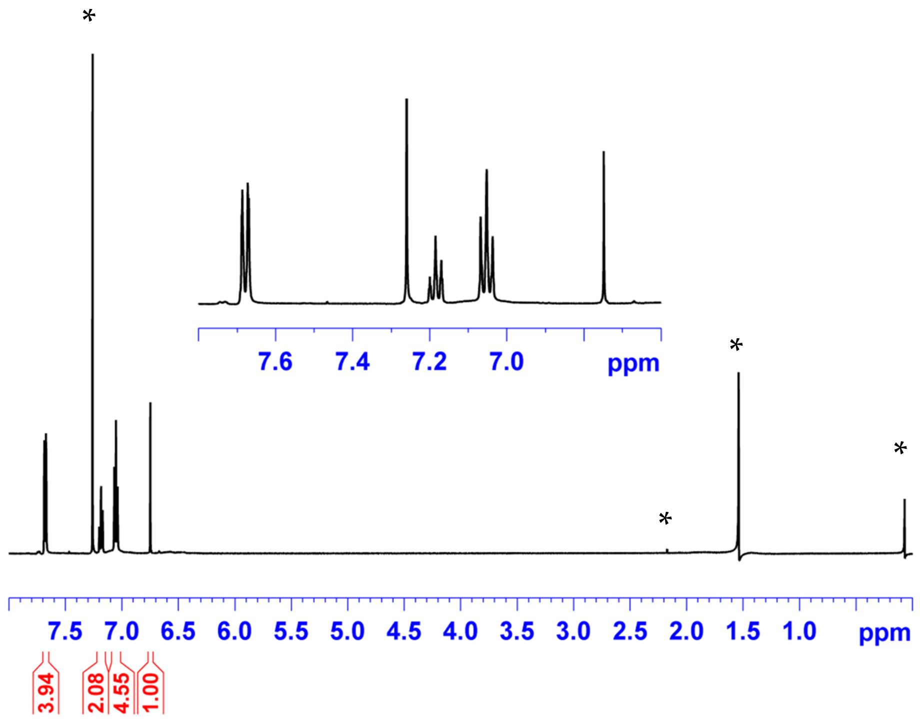

Figure S7. ^{1}H-NMR spectrum of **[Cu(μ-L5pz)]3** in $CDCl_3$ at room temperature (* marks solvents, water, and TMS peaks).

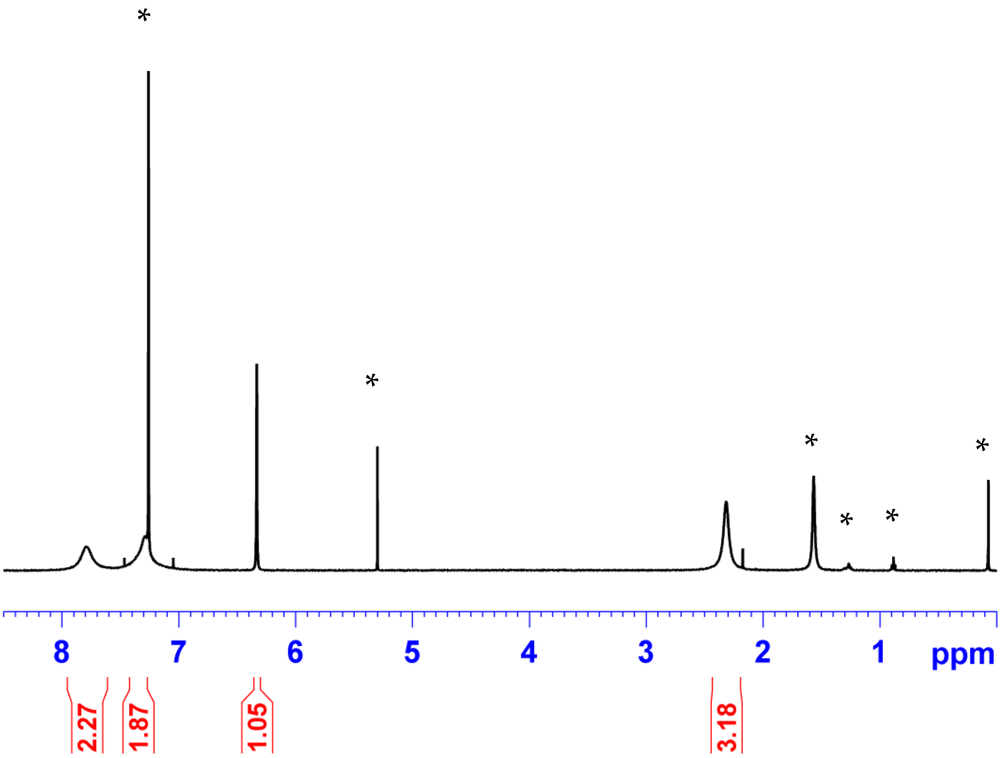

Figure S8. ^{1}H-NMR spectrum of **[Cu(μ-L6pz)]3** in $CDCl_3$ at room temperature (* marks solvents, water, and TMS peaks).

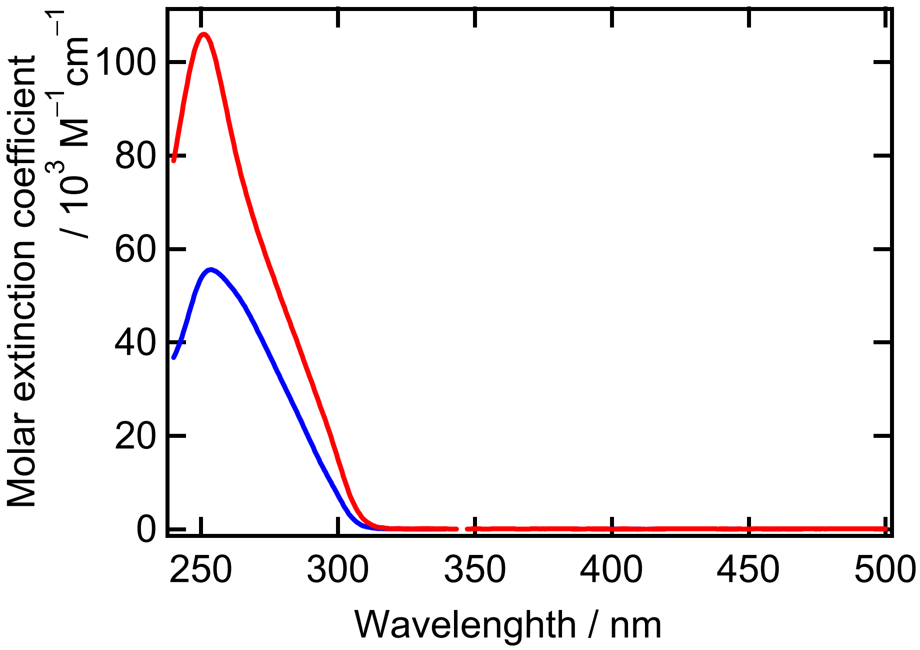

Figure S9. UV-Vis spectra of **[Cu(μ-L5pz)]$_3$** (red line) and **[Cu(μ-L6pz)]$_3$** (blue line) in dichloromethane at room temperature.

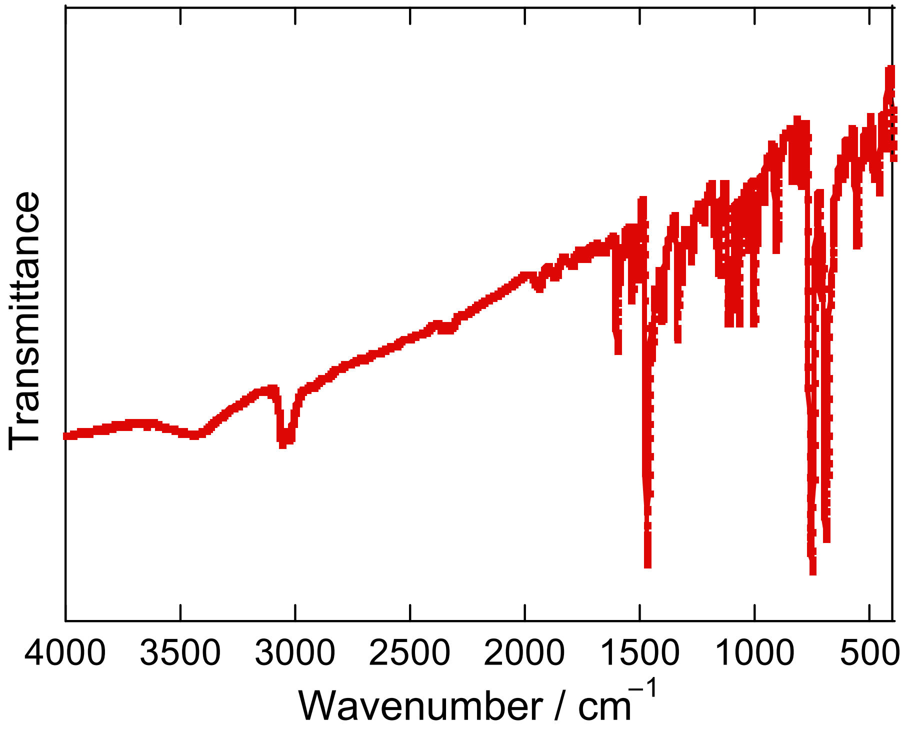

Figure S10. IR spectra of **[Cu(μ-L5pz)]$_3$** (red line) in KBr disk at room temperature.

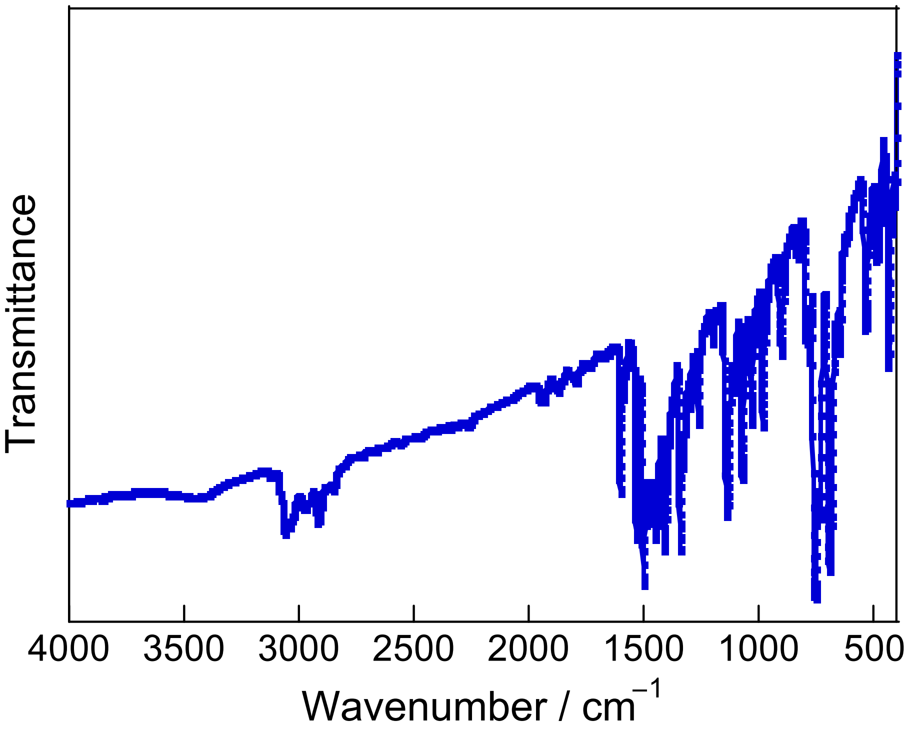

Figure S11. IR spectra of **[Cu(μ-L6pz)]$_3$** (blue line) in KBr disk at room temperature.

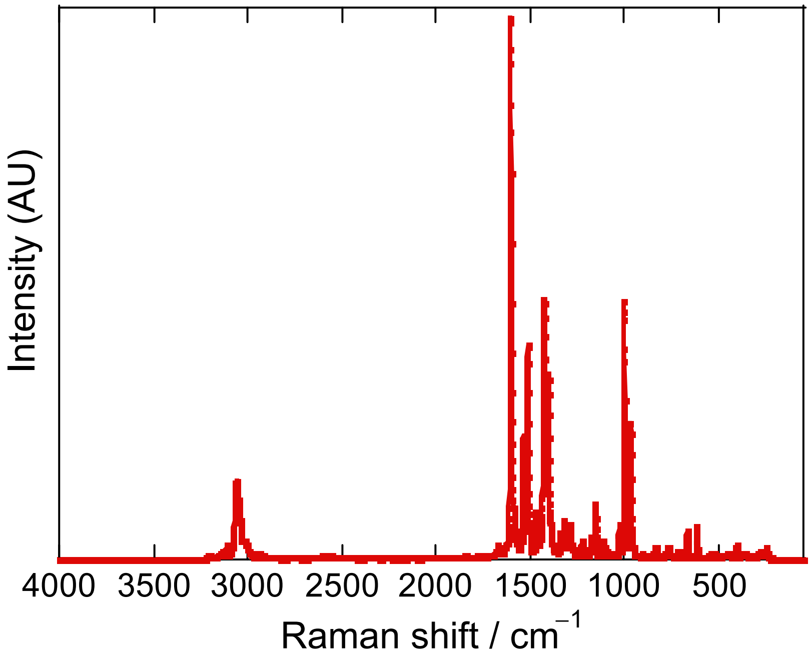

Figure S12. FT-Raman spectrum (600 mW Laser power) of **[Cu(μ-L5pz)]**$_3$ (blue line) solid at room. temperature.

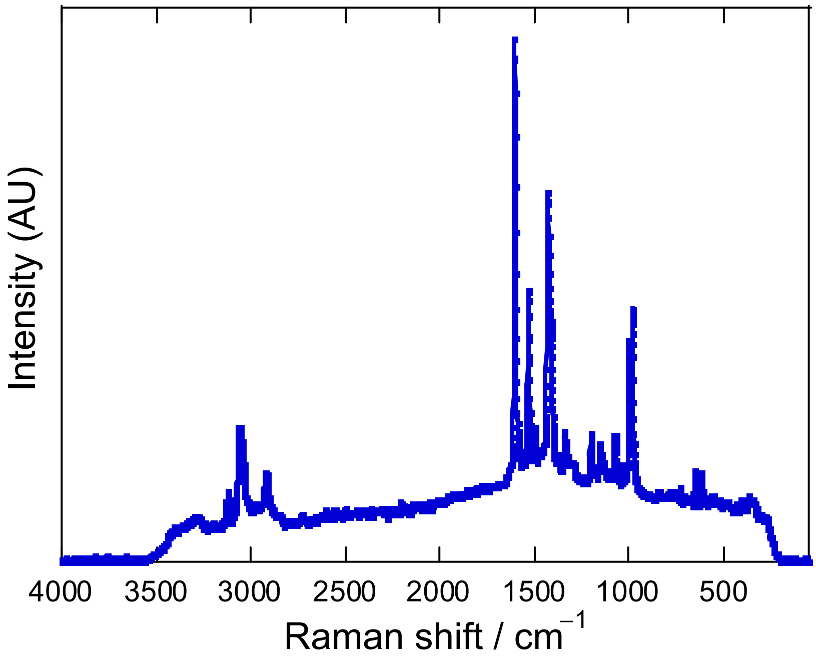

Figure S13. FT-Raman spectrum (600 mW Laser power) of **[Cu(μ-L6pz)]**$_3$ (blue line) solid at room. temperature.

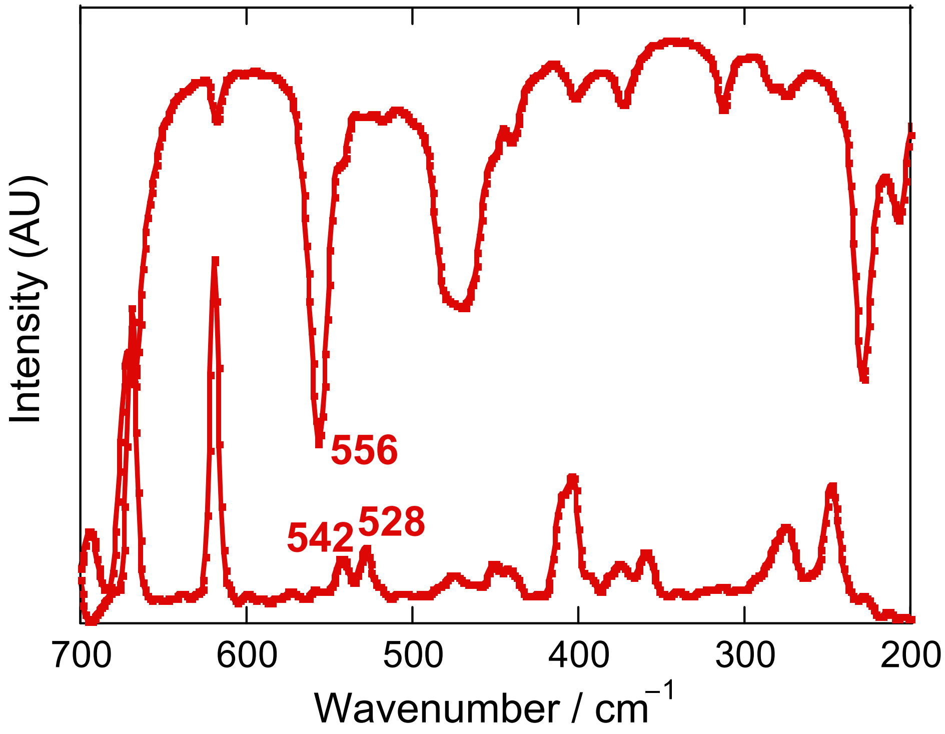

Figure S14. FT-Raman and far-IR spectra of **[Cu(μ-L5pz)]**$_3$ (red line) solid at room temperature.

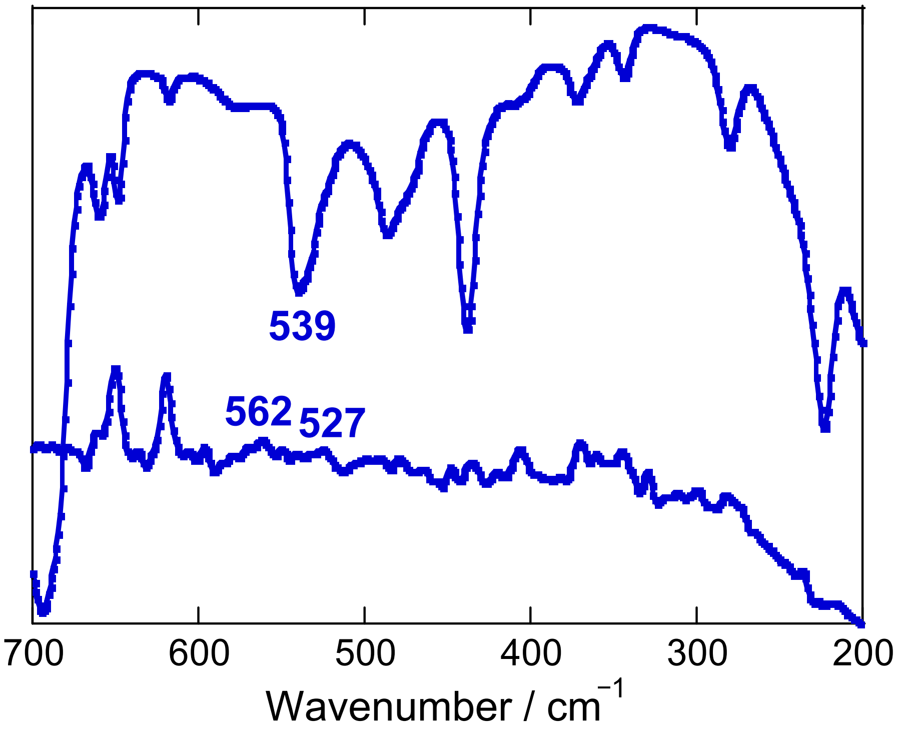

Figure S15. FT-Raman and far-IR spectra of **[Cu(μ-L6pz)]**$_3$ (blue line) solid at room. temperature.

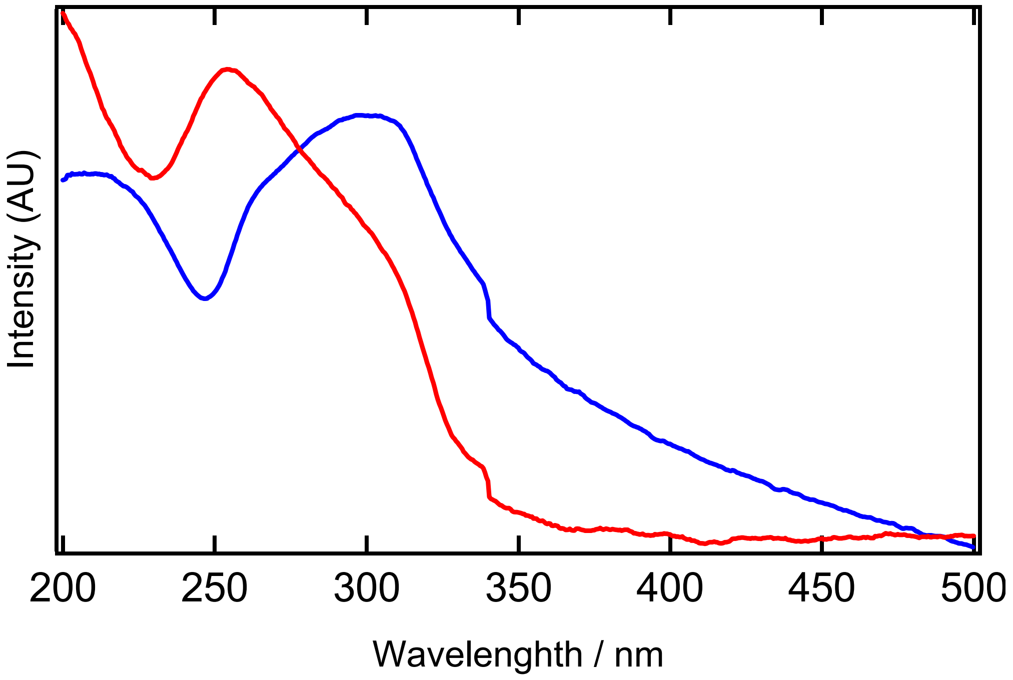

Figure S16. UV-Vis spectra of **[Cu(μ-L5pz)]**$_3$ (red line) and **[Cu(μ-L6pz)]**$_3$ (blue line) solid mull at room temperature.

(a) solid at 298 K

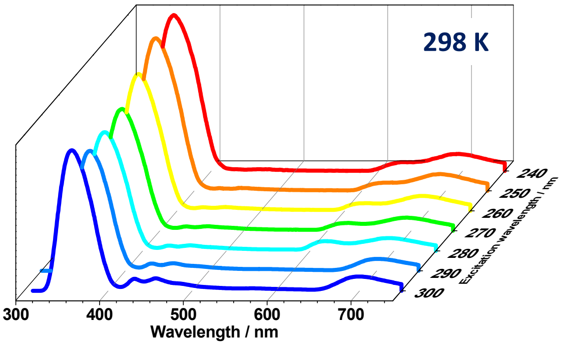

(b) solid 173 K

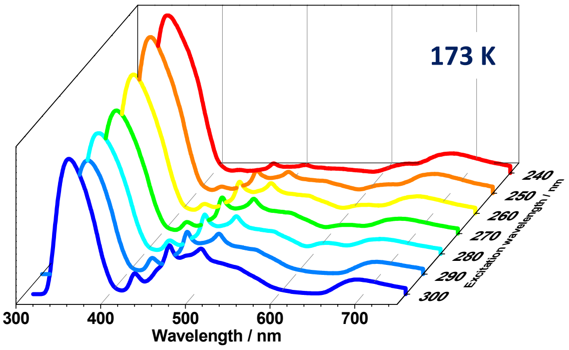

(c) solid at 83 K

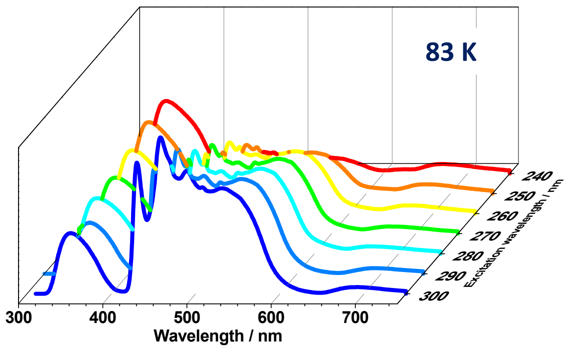

(d)

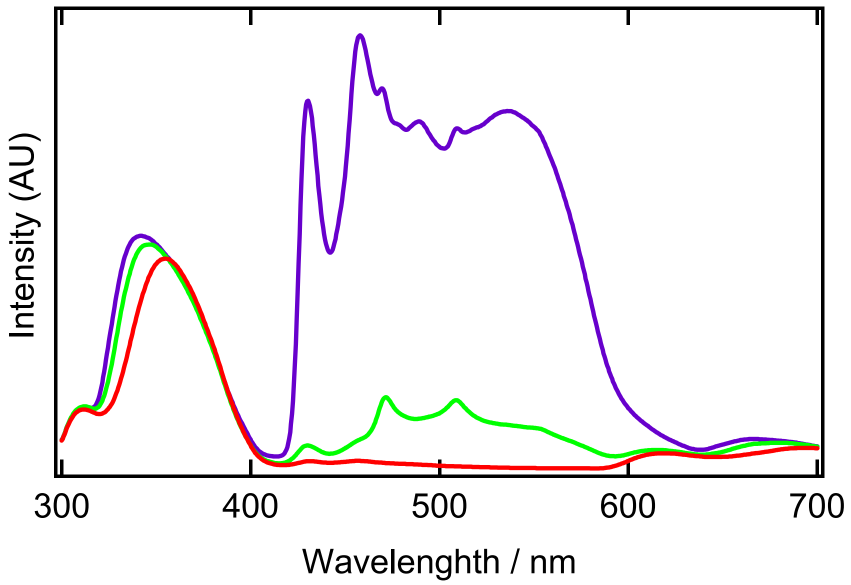

Figure S17. Photoluminescence spectra of **[Cu(μ-L5pz)]**$_3$ in the solid state with variable exciting wavelengths from 240 nm to 300 nm at three different temperatures ((a) 298 K, (b) 173 K, and (c) 83 K) and (d) temperature dependent photoluminescence spectra (83 K (violet), 173 K (green), and 298 K (red)) at 280 nm excitation wavelength.

(a) solid at 298 K

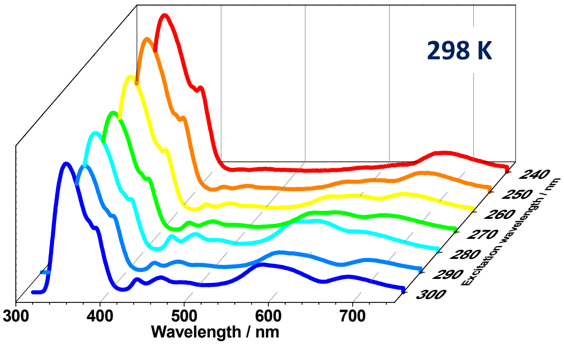

(b) solid 173 K

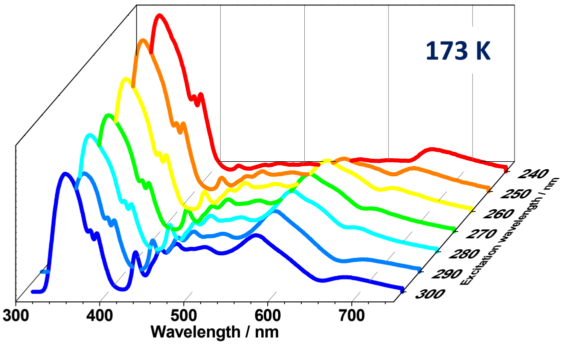

(c) solid at 83 K

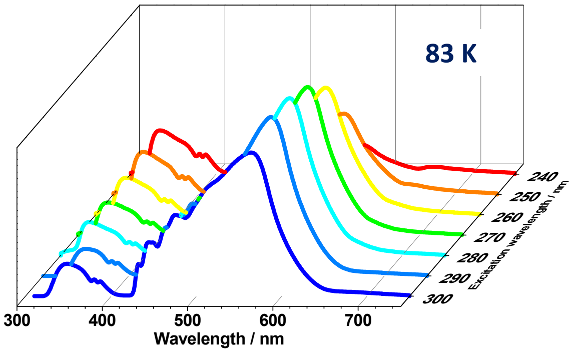

(d)

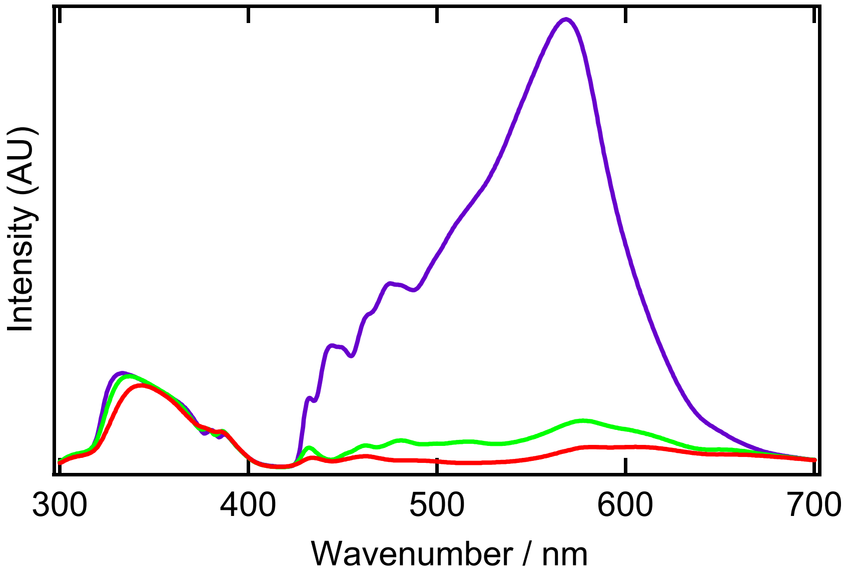

Figure S18. Photoluminescence spectra of **[Cu(μ-L6pz)]$_3$** in the solid state with variable exciting wavelengths from 240 nm to 300 nm at three different temperatures ((a) 298 K, (b) 173 K, and (c) 83 K) and (d) temperature dependent photoluminescence spectra (83 K (violet), 173 K (green), and 298 K (red)) at 280 nm excitation wavelength.

(a) solid at 298 K

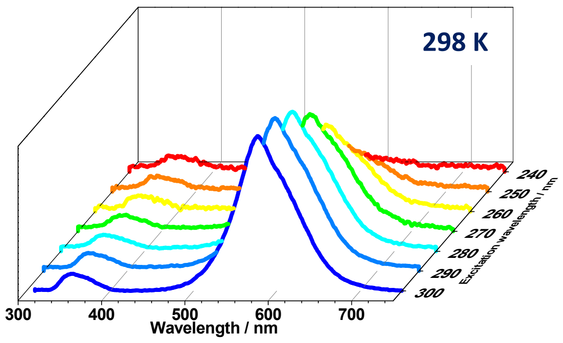

(b) solid 173 K

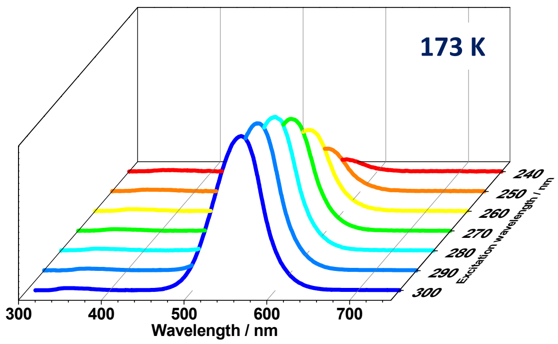

(c) solid at 83 K

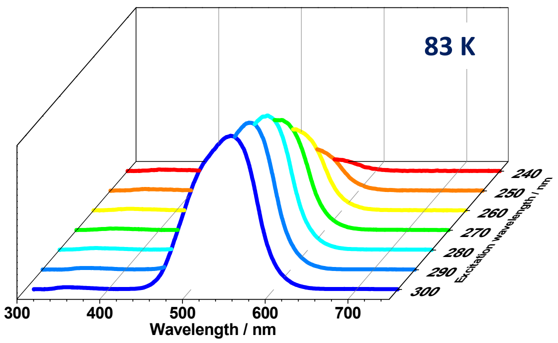

(d)

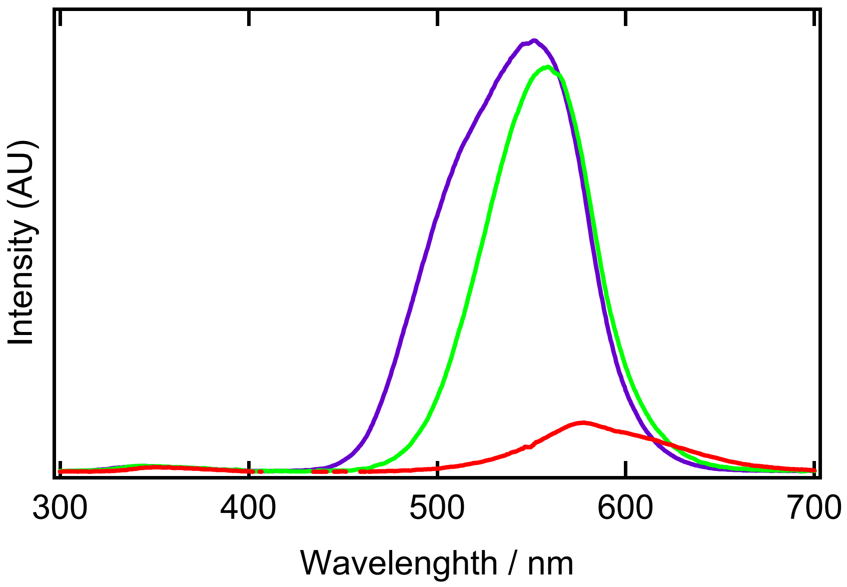

Figure S19. Photoluminescence spectra of [Cu(μ-3,5-iPr$_2$pz)]$_3$ in the solid state with variable exciting wavelengths from 240 nm to 300 nm at three different temperatures ((a) 298 K, (b) 173 K, and (c) 83 K) and (d) temperature dependent photoluminescence spectra (83 K (violet), 173 K (green), and 298 K (red)) at 280 nm excitation wavelength [2-3].

References

1. Raptis, R.G.; Fackler, J.P., Jr. Structure of tris(μ-3,5-diphenylpyrazolato-*N*,*N′*)tricopper(I). structural comparisons with the silver(I) and gold(I) pyrazolate trimers. *Inorg. Chem.* **1988**, *27*, 4179–4182.
2. Fujisawa, K.; Ishikawa, Y.; Miyashita, Y.; Okamoto, K. Crystal structure of pyrazolato-bridged copper(I) polynuclear complexes. *Chem. Lett.* **2004**, *33*, 66–67.
3. Fujisawa, K.; Ishikawa, Y.; Miyashita, Y.; Okamoto, K. Pyrazolate-bridged group 11 metal(I) complexes: substituent effects on the supramolecular structures and physicochemical properties. *Inorg. Chim. Acta* **2010**, *363*, 2977–2989.
